# Universal structural parameter to quantitatively predict metallic glass properties

**DOI:** 10.1038/ncomms13733

**Published:** 2016-12-12

**Authors:** Jun Ding, Yong-Qiang Cheng, Howard Sheng, Mark Asta, Robert O. Ritchie, Evan Ma

**Affiliations:** 1 Materials Sciences Division, Lawrence Berkeley National Laboratory, Berkeley, California 94720, USA; 2 Department of Materials Science and Engineering, Johns Hopkins University, Baltimore, Maryland 21218, USA; 3 Chemical and Engineering Materials Division, Oak Ridge National Laboratory, Oak Ridge, Tennessee 37831, USA; 4 Department of Physics and Astronomy, George Mason University, Fairfax, Virginia 22030, USA; 5 Department of Materials Science and Engineering, University of California, Berkeley, California 94720, USA

## Abstract

Quantitatively correlating the amorphous structure in metallic glasses (MGs) with their physical properties has been a long-sought goal. Here we introduce ‘flexibility volume' as a universal indicator, to bridge the structural state the MG is in with its properties, on both atomic and macroscopic levels. The flexibility volume combines static atomic volume with dynamics information via atomic vibrations that probe local configurational space and interaction between neighbouring atoms. We demonstrate that flexibility volume is a physically appropriate parameter that can quantitatively predict the shear modulus, which is at the heart of many key properties of MGs. Moreover, the new parameter correlates strongly with atomic packing topology, and also with the activation energy for thermally activated relaxation and the propensity for stress-driven shear transformations. These correlations are expected to be robust across a very wide range of MG compositions, processing conditions and length scales.

## Abstract (web summary)

Various known structural descriptors of metallic glasses have limitations in quantitatively predicting properties. Here authors define a physically-motivated measure and show it to correlate strongly with elastic properties, local structure and relaxation kinetics over a wide range of simulated compositions.

Intensive research is currently underway to understand the unusual structures and properties of metallic glasses (MGs)^1^,^2^,^3^,^4^,^5^,^6^,^7^,^8^,^9^. Despite relentless pursuit, quantitative structure-property relationships have not been successfully established thus far that are universally viable for MGs. This lags far behind conventional crystalline metals, for which many predictive relationships have been documented over the years, forming the cornerstones of materials science as a discipline. For example, explicit laws can be found in textbooks to predict the strength and plastic flow behaviour of an alloy. The key parameters involved in these relations are often the shear modulus, *G*, and the characters of defects, such as the dislocation density *ρ* and Burgers vector **b**. A simple example is the Taylor hardening law, giving the stress elevation due to dislocation accumulation as proportional to *ρ*^1/2^*G***b** (ref. 10).

Monolithic MGs, in contrast, do not have distinctly bifurcated lattices (with fixed *G*) and well-defined defects (for example, **b** and *ρ*). They are in fact invariably amorphous with no discernible microstructure^9^,^11^. Yet, widely different properties have been reported for MGs of different compositions^12^,^13^,^14^, or even MGs of the same composition but with different processing history^1^. *G* not only is much smaller than that of the corresponding crystal, but also varies with both the alloy composition and the processing history used to make the MG (quench rate, or ageing temperature and duration after the MG is made). In other words, now the property (such as *G*) is influenced by a wide distribution of local configurations that are variably defect-like inside the seemingly structure-less glass. A long-standing challenge is therefore to find a suitable indicator that can decipher structural differences distinguishing one MG from another or local regions that are inhomogeneous inside a given MG. The indicator also needs to have predictive power, allowing mathematical derivation of the properties from the structural state it represents.

To set the stage, let us first take a brief survey of several previously invoked structural indicators, the most common ones being the free volume^15^,^16^, configurational potential energy^7^, fictive temperature^17^,^18^, topological (for example, icosahedral) local order^9^,^19^, and atomic-level stresses^20^. These indicators have been useful for various analysis purposes, but all have their inherent limitations. For example, either the configurational potential energy^7^ or the fictive temperature^17^,^18^ can be used for representing the level of disorder in an MG state; but these state variables are not really descriptive of the structural origins *per se*. Such a metric, while meaningful to reflect the relative stability of different MG states at a given composition, is difficult to use to compare different compositions due to different and arbitrary reference states. The parameter most widely quoted in literature is perhaps the free volume, *υ*_f_. This concept was conceived for hard-sphere systems, and is thus deficient for describing metallic bonds characterized by much softer interatomic potentials^20^. The latter leads to ambiguous or inaccessible reference state (such as hard sphere or ‘ideal glass'^21^), and a low content of *υ*_f_ (refs 20, 22) that is distributed everywhere to all atoms. All these make *υ*_f_ difficult to identify, quantify and work with. Since an MG containing more free volume would have a larger average atomic volume, Ω_a_, the easily tangible Ω_a_ (or Voronoi cell volume, or the volume/density difference from the corresponding crystal) is often used to reflect the free volume content. Also problematic is that *υ*_f_ is insensitive to MG composition and processing history, and has recently been shown to be inadequate in correlating with property variations^23^,^24^ (several examples are given later).

Advances in dissecting the atomic packing topology have provided revealing details about the MG structures. Previous work has shown that in certain MGs, the characteristic coordination polyhedral motifs, such as full icosahedra (with Voronoi index <0, 0, 12, 0>) in Cu-rich Cu-Zr-based MGs, are not only the locally favoured structure but also play a key role in controlling properties such as relaxation dynamics^9^,^19^. However, different MGs have different preferred motifs, that is, different Kasper polyhedra, due to their different atomic size ratios^19^. Even motifs with the same Voronoi index do not have the same packing symmetry, and the chemical order is not explicitly revealed by the index. More recently, attention has also been paid to packing configurations that deviate the most from locally favoured structures: the ‘geometrically unfavoured motifs' (GUMs)^19^,^25^. When a local region contains a high content of GUMs, it can be among the most ‘liquid-like'. But there is no clear and easy boundary to demarcate which GUMs would be the ones that are actually activated to carry relaxation and deformation. Meanwhile, these topological descriptors are not amenable to use in mathematical equations. As such, a case can be made for the pressing need of a multiplex structural indicator, one that not only represents the extent of configurational disorder (including packing and excess volume), but also reflects the other functionally oriented state variables mentioned above.

To this end, this paper introduces a new parameter in the form of a volume-scaled (or density-normalized) vibrational mean square displacement (MSD). We show that this simple structural indicator, termed flexibility volume, is measurable both computationally and experimentally while enabling quantitative prediction of properties and exhibiting strong correlations with structural and kinetic details at the atomic scale. We also present simple physical arguments to motivate this parameter as a natural choice for characterization and comparison of MGs of different composition and processing history.

## Results

### Flexibility volume as a structural indicator of MGs

To establish such a parameter, we further postulate that it would be futile to define causal structure-property relationship based solely on the ‘static' structure of MGs. This is rooted in the nature of the MG structure. Different from crystals, the diverse short-range order and their medium-range correlations^19^, as well as the subtle variations between similar local configurations, make it practically impossible to predict with certainty the response of a local structure to external stimuli (thermal, mechanical, and so on), even when the static structure (the coordinates marking the relative positions of all atoms) is fully known. A more sensible approach, therefore, would be to observe how the atoms respond to the simplest excitations, and incorporate this trial information into an indicator of the (local) structural state. In other words, our approach is to ‘test the water', by driving the system to survey/sample its own potential energy profile in a way that can be easily implemented in simulations and measured in experiments. A tell-tale indicator can then be extracted that not only reflects the local static structure, but also gauges its susceptibility to dynamic activations such as thermal vibration and shear transformations. Such a structural parameter would serve better in conveying how the configurational state actually controls the properties.

**Figure 1:**
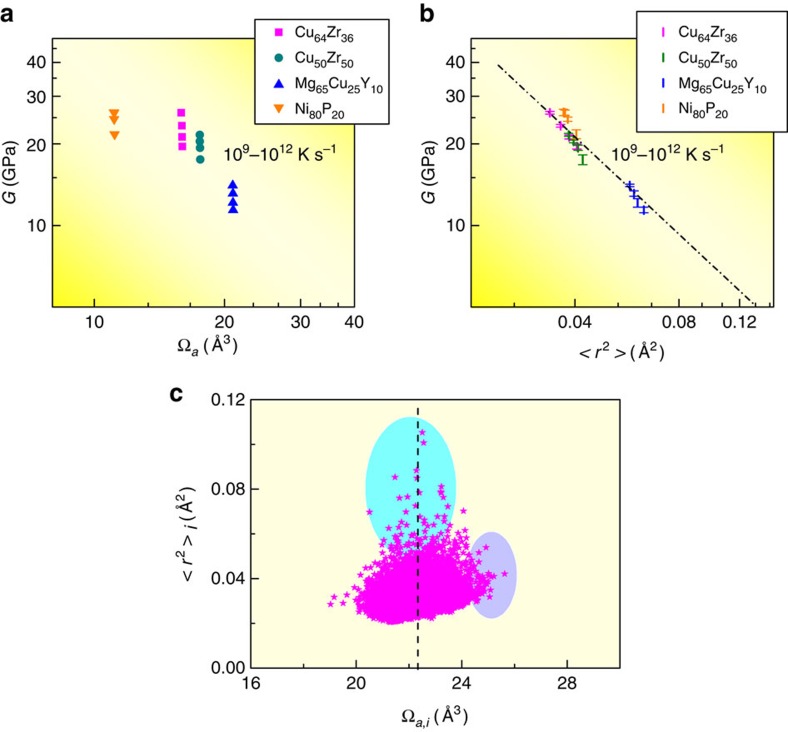
Vibrational mean square displacement in comparison with atomic volume. Molecular dynamics simulations of four representative MGs, including Cu_64_Zr_36_, Cu_50_Zr_50_, Ni_80_P_20_ and Mg_65_Cu_25_Y_10_, prepared using different cooling rates (Samples G1-G16 in Supplementary Table 1). (**a**) The change in shear modulus (*G*) is obvious for different cooling rates, but that in free volume (or Ω_a_) is not easily detectable. (**b**) In contrast, the obvious change in *G* appears to correlate with an obvious difference in ensemble-averaged vibrational MSD, at room temperature (with denoted error bar of standard deviation). This sensitive dependence reveals the important role of <*r*^2^> in reflecting the flexibility of atoms, although vibrational MSD alone does not quantitatively determine *G*, as discussed below. (**c**) The <*r*^2^>_*i*_ for the *i*th atom is not simply proportional to its atomic volume (Voronoi volume), Ω_a,*i*_. These two quantities are plotted and compared here, for each of the Zr atom in the Cu_64_Zr_36_ MG (Sample G1). The dashed line marks the system-average value of Ω_a_.

We next use a case study to illustrate what additional information is critically missing when correlating with properties, by examining the correlation between *G* and Ω_a_ as an example of the structure-property relations. The choice to discuss *G* is because it is widely regarded as a key baseline property for MGs. Specifically, *G* controls the energy barrier^7^ for relaxation (and shear flow), as shown for example in the cooperative shear model of Johnson and Samwer^26^, and is also strongly dependent on glass configuration (and hence on processing history). Once *G* is known, a number of important MG properties can be deduced from semi-empirical correlations, including the glass transition temperature *T*_g_, the yield strength, the energy barrier height for relaxation^13^,^26^,^27^,^28^, the change of fracture toughness upon ageing^29^ and even fragility of the corresponding supercooled liquid^30^,^31^. Examples of known empirical correlations with *G* are shown in Supplementary Fig. 1. As for Ω_a_, it can be taken as a reflection of the content of the commonly cited free volume, as mentioned earlier. So the *G* versus Ω_a_ relation would be a suitable case study to test if (free) volume alone would suffice for a robust structure-property relationship. Previous experimental data have shown that in MGs *G* (as well as the bulk modulus *B*) has an approximate scaling relationship with Ω_a_^27^ (or average inter-atomic distance^13^): the smaller the Ω_a_, the larger the *G* and *B*, as shown in Supplementary Fig. 2. However, this is only an overall trend; the scatter is obvious even when the *G* values are plotted on a logarithmic scale (Supplementary Fig. 2). More importantly, the data fitting could be done in multiple ways, but any empirical equation would lack a fundamental physical basis. Therefore, one cannot derive quantitatively a one-to-one correspondence from such plots. In addition, our own tests in Fig. 1a show that when MGs at a fixed composition (four examples) are produced with cooling rates differing by three orders of magnitude from the parent liquid, *G* changes markedly, but the corresponding change in Ω_a_ is barely detectable. All these demonstrate that Ω_a_ is quite insensitive to the configurational state^20^, and motivate again the need for a better parameter, in lieu of the free volume, to achieve our goal of a quantitative relationship with predictive power for MG solids.

To observe what other information would be desirable, let us examine the correlation with a dynamical parameter, the vibrational MSD, <*r*^2^>. (An example of vibrating atomic motifs can be seen in Supplementary Movie 1.). The vibrational MSD evaluated for the same four different MG systems prepared with different cooling history (hence different configurations) is plotted versus *G* in Fig. 1b. We observe that <*r*^2^> not only exhibits obvious configurational dependence, comparable to that for *G* (the two each span a sizable range), but also brings together different MG systems onto a common scaling relationship with *G*. This correlation persists when many more MGs with different compositions and prepared at different cooling rates are included, as shown in Supplementary Fig. 3. What <*r*^2^> adds is information about the flexibility of the local structural environment, obtained by dynamically probing the vibrational degree of freedom, reflecting the curvature at the basin of the local potential energy landscape (PEL). Such information is especially important in dealing with cases where the absolute magnitude of the free volume alone does not explain or control the atomic behaviour^23^,^24^. This approach is akin to the local Debye-Waller factor previously utilized to study supercooled liquids^23^,^32^,^33^,^34^.

To show that the vibrational MSD is not merely another way of measuring atomic volume, in Fig. 1c we plot these two quantities for each and every (the *i*th) Zr atom in a Cu_64_Zr_36_ MG. Most atoms reside in the magenta blob, displaying no strong correlation. Moreover, we observe that the cyan region, in which atoms have the highest <*r*^2^>_*i*_, does not have any overlap with the violet region for atoms having the largest atomic volume, Ω_a,*i*_. In other words, atoms can exhibit high <*r*^2^>_*i*_ without having extraordinary Ω_a,*i*_, and large Ω_a,*i*_ does not necessarily mean large <*r*^2^>_*i*_. This observation is in fact not surprising. As a thought experiment, consider a case when the local volume is not large (for example, around the average in Fig. 1c). This volume can distribute non-uniformly around the atom (strong shape anisotropy, to be further discussed later), leaving an easy avenue that is dynamically accessible for vibration (and presumably also relaxation to produce non-affine displacement). Also, some atoms with relatively large Ω_a,*i*_ may be caged in highly ordered and rigid coordination polyhedra such that their <*r*^2^>_*i*_ can be well below the average. We thus desire to also incorporate the information from <*r*^2^>, rather than relying on Ω_a_ alone, to assess how flexible the atoms actually are at a given temperature *T*, in their response to stimulus.

Note that the vibrational MSD alone^35^ is also not sufficient to enable a universally quantitative prediction of MG properties: obvious scatter is again present in Fig. 1b and Supplementary Fig. 3, even on a log-log scale. The new indicator, termed ‘flexibility volume' (*υ*_flex_), is therefore constructed as


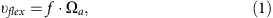


where 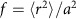 brings in the critical information from the vibrational MSD via the Lindemann ratio, previously employed to probe liquid viscosity^35^,^36^,^37^ or solid-liquid transition^38^,^39^. The normalization by *a*^2^, where 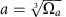 is the average atomic spacing, also renders *f* dimensionless. On the one hand, *υ*_flex_ combines the information of both atomic volume and vibrations, thus it can be thought as the volume-scaled vibrational MSD; on the other hand, it has the unit of volume, akin to free volume, but contains dynamics information. To paraphrase equation (1), the free volume is supposed to reflect the elbow room, ‘free' to redistribute for dilatation and relaxation, so the flexibility would scale with it, as is usually assumed. But *f* also influences the flexibility effectively achievable, as <*r*^2^> signals the wiggle room actually accessed, now sensed via the thermal vibrational probe at a given temperature. In other words, the product of the two, *f* and Ω_a_ together, reflects the space actually afforded by the (local) structural configuration in dynamic response. Note that Ω_a_ is two orders of magnitude too large to quantitatively represent the free volume, which should be of the order of 1% of Ω_a_ (refs 20, 22). The *f* factor brings down its magnitude to the level of free volume, as 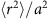 is of the order of a fraction of 1% at ambient temperature. But now *υ*_flex_ is encoded with information about actual flexibility.

We stress here that, above all, the most important reason to define flexibility volume as in equation (1) is the equation below (see derivation in Supplementary Note 1), which illustrates that when *υ*_flex_ is defined this way, a new volume parameter emerges that universally and deterministically controls *G* based on the Debye model^35^,


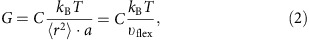


where the constant 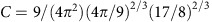. This derivation predicts that at a given temperature *T* (for example, room temperature), a single indicator, *υ*_flex_ by itself, can predict the *G* for all MGs. The message is then that the new flexibility volume indicator is not merely an equivalent substitute of other volume parameters, (Ω_a_, *υ*_f_ and so on), nor a fudge factor in equations. Rather, the *υ*_flex_ is unambiguously quantified and incorporates dynamics information, making it a conceptual advance over all previous static structural descriptors. In the meantime, *υ*_flex_ is a truly property-controlling volume parameter: it is the proper volume variable needed in the denominator if one normalizes the energy *k*_B_*T* in the numerator to arrive at *G* (energy density per unit volume) in equation (2). *G* could also be pictured as a mechanical metric of the flexibility of motion.

### Quantitative verification of the universal *υ*_flex_- *G* relation

**Figure 2:**
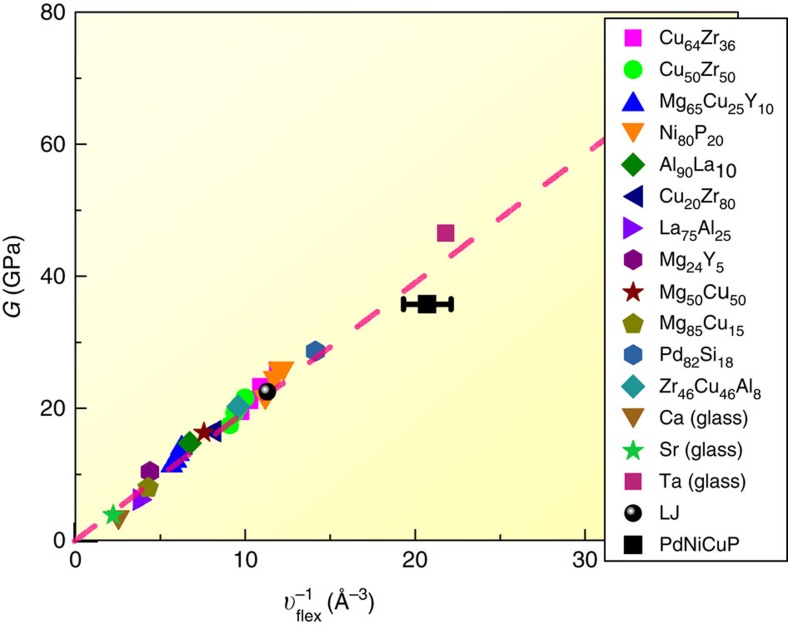
Correlation between shear modulus and flexibility volume in metallic glasses. The values of *G* and *υ*_flex_ are computed for 32 MGs at 300 K (see Supplementary Table 1 for different cooling rates and compositions, including an L-J glass). The dashed straight line is the predicted correlation derived in equation (2). The data point for the Pd-Ni-Cu-P MG is from an experimental measurement of both *G* and *υ*_flex_ (from MSD) for a given sample^50^ (see discussion in Supplementary Note 2).

Both *υ*_flex_ and *G* can be measured computationally in model MGs. For each MG, we evaluated the *υ*_flex_ in equation (1) for each individual atom (that is, *υ*_flex,*i*_), using <*r*^2^>_*i*_ obtained on short time scales when the MSD is flat with time and contains the vibrational but not the diffusional contribution (see Methods). The magnitude of *υ*_flex,*i*_ (of the order of 0.1 Å^3^) is a fraction of the expected free volume (typically of the order of 1% of the space occupied by the atom, Ω_a,*i*_, which is 10∼20 Å^3^ in Fig. 1a). Over the past ten years we have been developing embedded atom method interatomic potentials for a number of model systems, including Cu-Zr-Al, Mg-Cu-Y, Pd-Si, Ta (refs 40, 41, 42, 43). We are thus able to use MD simulations (see Methods) to acquire data for a variety of MG alloy systems, including a wide range of compositions in each system, and different structural states reached at each composition by using a range of different cooling rates for MG preparation from the parent liquid. The large database, tabulated in Supplementary Table 1, has enabled us to quantitatively test the universal *G***-***υ*_flex_ relationship in equation (2) for MGs. Figure 2 summarizes the sample-averaged *υ*_flex_ and *G*, computed for ∼32 different MGs at room temperature. These data sets conform remarkably well to the predicted relationship in equation (2), which is the straight line in Fig. 2. Supplementary Fig. 4 also plots data of *υ*_flex_ versus *G* obtained at different simulation temperatures, to demonstrate the general validity of equation (2). The quantitative relationship established over a wide range of values for *υ*_flex_ and *G* in these figures is impressive, demonstrating the power of the *υ*_flex_ in normalizing the vibrational MSD to unify so many different MG types and enable a universal correlation. Note that *G* of simulated MGs is computed using the fluctuation method^44^, which is theoretically derived from the framework of lattice dynamics^31^. But compared with the theory of lattice dynamics^31^, *υ*_flex_ is much easier to work with both computationally and experimentally. Also, the systematic data set confirms the general, and perhaps even surprising, applicability of the Debye model to amorphous metals. As far as MGs are concerned, *υ*_flex_ outperforms by far the free volume, which, even if its absolute value is known, cannot be used to directly calculate any particular property. The advantages of *υ*_flex_ will be further illustrated and advocated in the following.

### Flexibility volume correlates strongly with local structure

**Figure 3:**
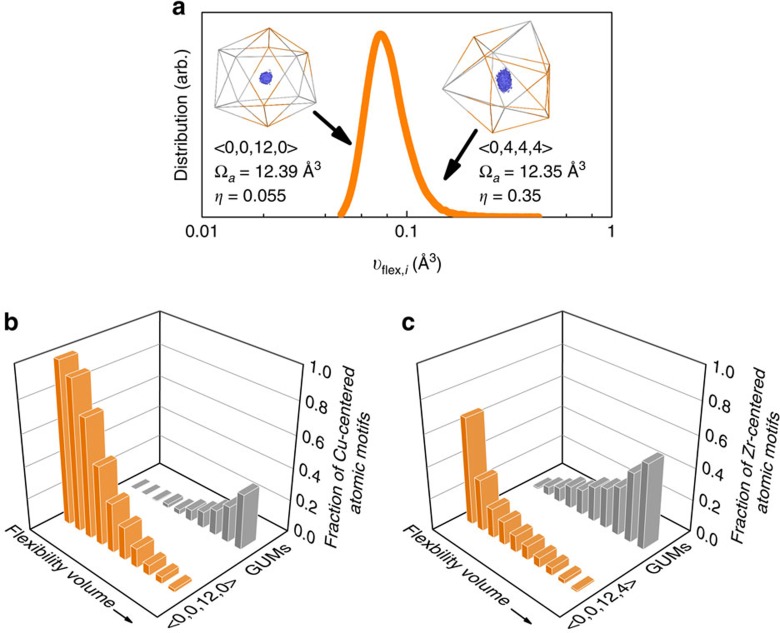
Flexibility volume correlates strongly with local atomic packing structure. (**a**) Distribution of flexibility volume (*υ*_flex,*i*_) in a Cu_64_Zr_36_ MG (Sample G28). The insets show two Cu atoms in this distribution. These two atoms (each at the centre of a Voronoi polyhedron as indexed above) have almost the same atomic volume (Voronoi volume), but the more anisotropic case (higher *η*) has a value for *υ*_flex,*i*_ twice as large as that in the more regular full icosahedron. The bi-coloured lines connect nearest neighbours (grey for Zr and gold for Cu). The central blue region represents the maximum volume sampled during the simulation by the centre of mass of the vibrating Cu atom. In (**b**) (or (**c**)), Cu (or Zr) atoms in this Cu-Zr MG are first sorted by their flexibility volume (from low to high), and then divided into ten groups each containing 10% of all Cu (or Zr) atoms. The fraction of Cu-centred <0, 0, 12, 0> (or Zr-centred <0, 0, 12, 4>) and GUM clusters present in these ten groups is then compared. For the 10% of the atoms with the lowest flexibility volume, almost all of the Cu atoms are in <0, 0, 12, 0> (and Zr in <0, 0, 12, 4>) clusters, whereas most of the 10% atoms with the highest flexibility volume are in GUMs.

Next, we demonstrate how well *υ*_flex,*i*_ correlates with local structure, to further establish the flexibility volume as a revealing indicator of the structural state of the MG on atomic levels. Firstly, we reiterate that *υ*_flex,*i*_ is different from the local volume (for example, Ω_a,*i*_). The *υ*_flex,*i*_ distribution in the Cu_64_Zr_36_ MG is shown in Fig. 3a, which is close to a Gaussian distribution (this is shown for other MGs in Supplementary Fig. 5, where *υ*_flex,*i*_ is seen to span two orders of magnitude). Shown in the inset is an example, where we compare the two Cu atoms each at the centre of its coordination polyhedron. The more anisotropic case (the one with Voronoi index <0, 4, 4, 4> and smaller Ω_a,*i*_) exhibits a flexibility volume obviously larger than the more isotropic case (<0, 0, 12, 0>). This reaffirms the message in Fig. 1c; atoms with high *υ*_flex,*i*_ do not necessarily have large Ω_a,*i*_, and vice versa. More discussions are presented in Supplementary Fig. 6, to confirm that *υ*_flex,*i*_ indeed scales with the degree of vibrational anisotropy, *η* (see Methods), which is therefore a parameter that promotes flexibility. Supplementary Fig. 6e–f further illustrates that GUMs are more likely to have higher *η*; as expected the increased degree of distortion in the coordination polyhedra corresponds to higher anisotropy. In this regard the advantage of *υ*_flex,*i*_ over Ω_a,*i*_ is obvious; the latter is indiscriminate about this shape or anisotropic spatial distribution, thus missing important structural information that affects the flexibility.

Figure 3a also demonstrates that *υ*_flex,*i*_ is sensitively correlated with the atomic-level packing topology of the *i*th atom. Here two representative Cu-centred atomic motifs, with the Voronoi index of <0, 0, 12, 0> and <0, 4, 4, 4>, respectively, are displayed as an example. The Cu-centred clusters with the Voronoi index of <0, 0, 12, 0> (full icosahedra) are the most stable atomic motif in Cu_64_Zr_36_ as illustrated before^45^, and they are expected to have small *υ*_flex,*i*_. In comparison, atomic motifs with the index of <0, 4, 4, 4> belong to the category of GUMs and are expected to contain more *υ*_flex,*i*_. This contrast in *υ*_flex,*i*_ is indeed observed in Fig. 3a. To statistically establish the connection between *υ*_flex,*i*_ and atomic packing topology, systematic data are presented in Fig. 3b,c; the locally favourable motifs, Cu-centred <0, 0, 12, 0> and Zr-centred <0, 0, 12, 4>, correspond to the minimum *υ*_flex,*i*_, which is in stark contrast to GUMs, which tend to have large *υ*_flex,*i*_. Another such example is given for the Al_90_La_10_ MGs in Supplementary Fig. 7. Supplementary Fig. 8 also includes plots that demonstrate the correlation of *υ*_flex,*i*_ with the configurational potential energy (and hence with the fictive temperature).

### Strong correlation with local relaxation events

We now address how well *υ*_flex,*i*_ correlates with several other important properties, on multiple levels and length scales. Of particular interest are the localized soft vibrational modes, the energy barrier for thermally activated relaxation events and the stress-driven elementary shear transformations. For the former, a connection was uncovered earlier between the local packing structure and the quasi-localized low-frequency vibration modes (that is, the soft spots where a nanometer-sized region contains a high content of atoms that participate strongly in soft modes)^25^. As demonstrated in Supplementary Fig. 9a–b, a very strong statistical correlation is clearly seen between *υ*_flex,*i*_ and the participation ratio in soft modes (whereas no correlation is apparent with excess atomic volume, as seen in Supplementary Fig. 9c–d). This is expected since they both have the same origin in atomic vibration. We can therefore use high *υ*_flex,*i*_ in lieu of high participation ratio to embody the soft spots. This removes several shortcomings associated with soft mode analysis. The soft spots were identified based on a pre-selected cut-off vibrational frequency (for example, arbitrarily choosing the 1% lowest frequency), and the participation of atoms in these soft modes is evaluated on a relative basis^25^. This makes it difficult to decide which soft spots are truly eventful, in terms of being actually activated in relaxation. There is also no quantified measure of their contributions to the overall MG properties. Moreover, it is not feasible to compare the soft spots in different samples. In comparison, *υ*_flex_ is universal and easier to use, and it quantitatively scales with *G*. One can now use *υ*_flex_ to directly compare different MGs, and explain the spatial heterogeneity of mechanical properties mapped out for different local regions.

**Figure 4:**
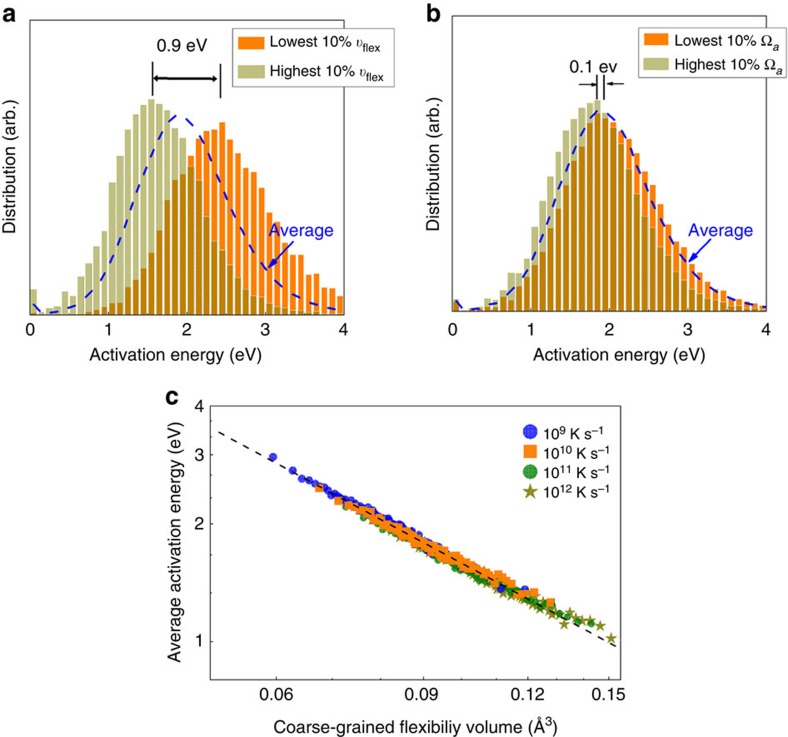
Flexibility volume correlates strongly with thermally activated relaxation events. Distribution of activation energy in a Cu_64_Zr_36_ MG (Sample G28, with 10,000 atoms and the cooling rate of 10^9^ K s^-1^) characterized using ART nouveau. The activated relaxation events are for (Cu and Zr) atoms in the centre of their coordination polyhedra. The blue line is for the distribution of activation energy in the entire sample, whereas (**a**) shows the two groups with the highest and lowest 10% of the values for the flexibility volume (*υ*_flex,*i*_), and (**b**) is for the two groups with the highest and lowest 10% of the values for the atomic volume (Ω_a,*i*_) . For (**a**) and (**b**) both Cu and Zr atoms in the distribution are counted to avoid possible biases due to different chemical elements (that is, for the 10% of the atoms with the highest values of *υ*_flex,*i*_, we select both the top 10% Cu and the top 10% Zr atoms). Here each curve is normalized by the total number of activated events sampled by the entire group of atoms involved in the distribution. While the two groups almost overlap in the case of Ω_a,*i*_, the bifurcation between the two groups is obvious in the *υ*_flex,*i*_ case, with the two peaks separated by 0.90 eV. (**c**) All the atoms are sorted based on coarse-grained values of *υ*_flex,*i*_ (see text), into bins each containing 1% of the atoms. An average activation energy is then calculated for the atoms in each bin, and plotted to demonstrate the strong correlation with the coarse-grained flexibility volume. The Cu_64_Zr_36_ MG samples used in this plot were produced at several cooling rates.

The next property to correlate with is the activation energy barrier for thermally activated relaxation events (*β* processes), which can be monitored using the activation-relaxation technique (ART nouveau) in MD simulations^46^,^47^,^48^ (see Methods). From the PEL perspective, the *α* process can be pictured as the transitions between the deep ‘metabasins', whereas the *β* process refers to the elementary hopping event between the ‘sub-basins' confined within a metabasin. These processes are related to many important properties (for example, glass transition, deformation, ageing, diffusion) of MGs. Figure 4a shows the distribution of activation energy in a Cu_64_Zr_36_ MG, for atoms having the lowest 10% and highest 10% *υ*_flex,*i*_. Atoms (at the centre of the local activation events) with lower flexibility (that is, smaller *υ*_flex,*i*_) are expected to need more energy to overcome the activation barrier, and vice versa. As seen in Fig. 4a, there is a major difference of ∼0.9 eV between the two peak positions for the two groups with the lowest and the highest 10% *υ*_flex,*i*_. We also obtained coarse-grained *υ*_flex,*i*_ by averaging over the centre atom and its nearest-neighbour atoms, because activated events usually involve a small group of atoms (on the order of a dozen) rather than one single atom^48^. The resulting separation of the two peaks is even wider (as shown in Supplementary Fig. 10). As shown in Fig. 4c, the correlation between the coarse-grained *υ*_flex,*i*_ and bin-averaged activation energy (see figure caption) is particularly strong, unifying samples produced with various cooling rates. This clearly demonstrates that *υ*_flex,*i*_, while incorporating the fast dynamics information based on vibrational (phonon) behaviour, is an effective indicator for correlating with the slow dynamics of *β* relaxation, in particular its activation energy barrier. The same cannot be said for free volume; in Fig. 4b we observe that the atoms with the highest and the lowest atomic volume do not exhibit obviously different activation energy barriers. The distribution curves of the two groups almost overlap with each other, displaying a small difference of only ∼0.10 eV in peak positions. This once again points to the inadequacy of Ω_a_ (or *υ*_f_) in correlating with dynamic properties.

**Figure 5:**
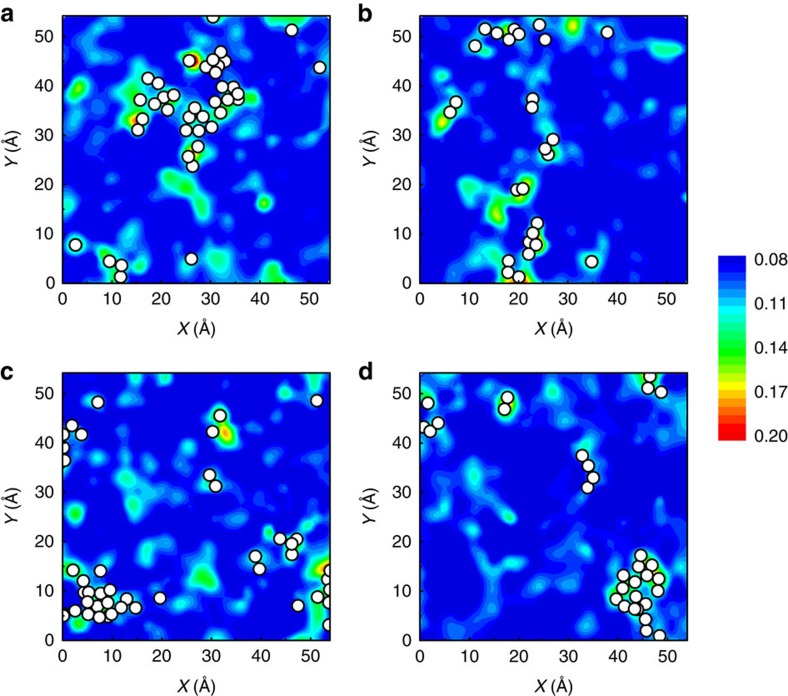
Strong correlation between *υ*_flex,*i*_ and shear transformations. Contoured maps show the spatial distribution of flexibility volume *υ*_flex,*i*_ (see sidebar) in the Cu_64_Zr_36_ metallic glass (Sample G28). Four slabs (**a**–**d**) are sampled for illustration purposes and each has a thickness of 2.5 Å. White spots superimposed in the maps mark the locations of atoms that have experienced the most (top 5%) accumulative non-affine displacement (*D*^2^_min_), upon athermal quasi-static shear of the simulation box to a global strain of 5%. Note that not all such regions would undergo shear transformation for a particular loading. This is reasonable because apart from the intrinsic flexibility of the local configurations, the stress field (tensor) is another (extrinsic) factor that will influence the response of the atoms.

Finally, we examine the response to the stress stimulus. Different from the thermally activated *β* processes, now the shear transformations are essentially stress-activated and they are the fundamental processes underlying the anelastic deformation; their percolation will eventually lead to *α* processes which correspond to macroscopic plastic flow leading to shear band formation. Figure 5 shows that *υ*_flex,*i*_ is also a very effective indicator of the propensity for shear transformations in MGs. Specifically, here athermal quasistatic shearing^49^ was applied to induce atomic rearrangement in a Cu_64_Zr_36_ MG, and the shear transformations were tracked by monitoring the non-affine displacement *D*^2^_min_ (ref. 25). The contoured maps of the spatial distribution of *υ*_flex,*i*_ are then compared/superimposed with the top 5% local motifs that have experienced the most accumulative non-affine strains, after a global strain (for example, 5%). The clear correlation in Fig. 5 establishes that under externally imposed stresses, shear transformations have a high propensity to originate from those regions with the highest flexibility volume. In contrast, such a correlation is absent with the variation of local excess atomic volume, as shown in Supplementary Fig. 11.

Before closing, we note that one can experimentally determine the flexibility volume of an MG, by measuring the vibrational MSD or the Debye temperature. The experimental measurement of the <*r*^2^>_*i*_ or the Debye-Waller factor at local and atomic scale must await future development of (sub)nanoscale probes, but on macroscopic samples measurements of the averaged values of these properties can be performed using several methods, including inelastic neutron scattering, extended X-ray absorption fine structure and X-ray/neutron diffraction (see Supplementary Note 2 for a detailed discussion on these methods and references). Such scattering characterization experiments^50^,^51^,^52^ have been reported previously, but they rarely measured *G* of the same MG sample. A data point was found from ref. 50, which has been added into Fig. 2 to support the MD-confirmed *υ*_flex_-*G* relation.

## Discussion

For MGs at temperatures well below glass transition, the advantages of flexibility volume over previous structural descriptors are multifold, as summarized below in eight respects. First, the *υ*_flex_ is clearly defined, from the atomic level and up, making it a simple and yet quantitative structural parameter. Second, the absolute value of *υ*_flex,*i*_ is directly measurable, both computationally and experimentally, incorporating the readily known atomic volume and the familiar vibrational MSD (not either one alone). Third, *υ*_flex,*i*_ is a universal indicator that enables comparison of various MG states (and properties) at different compositions and processing conditions, mapping all of them onto a common metric and reference (for example, the wide range of *υ*_flex_ and *G* for over 30 MGs in Supplementary Table 1 and Fig. 2). Fourth, the effects of anisotropic distribution of the accessible volume, as well as of local packing environment and chemical interaction between neighbouring atoms, are all included in, or reflected by, the flexibility volume. Fifth, as an advance over static structural descriptors it also incorporates dynamics survey information obtained from probing the landscape, akin to Debye-Waller parameter used before for viscosity and dynamic heterogeneity in liquids. From these latter two aspects, a collection of factors is now replaced by a single workable metric *υ*_flex_, which is then expected to connect strongly to MG behaviour, as indeed seen in the next three areas. Sixth, *υ*_flex_ is actually the ‘tell-tale' structural parameter deterministic of shear modulus, equation (2). Such a quantitative correlation was not possible for all standard structural parameters, including free volume and fictive temperature (and even the MSD alone, which was hypothesized^35^ to correlate with shear modulus but not demonstrated). Specifically, our extensive and systematic data set establish that MGs can be treated as normal Debye solid, with *υ*_flex_ as the proper variable to quantitatively link the vibrational behaviour with elastic constants. Moreover, through *G* and its correspondence with other state variables^7^,^13^,^26^,^27^,^29^, *υ*_flex_ serves to provide a common underpinning that predicts the various properties originating from the configurational state. For example, increasing the quench rate or ageing temperature around *T*_g_ of an MG would impart a higher *υ*_flex_ (for example, the Cu-Zr case in Fig. 1), which then quantitatively predicts a lowered *G* that reduces the barrier for shear flow, and hence an exponentially increased participation probability in shear transformations and consequently fracture toughness^29^. Seventh, *υ*_flex_ exhibits strong correlation with the participation in low-frequency soft vibrational modes (soft spots), and more usefully with slow dynamics such as (energy barrier for) thermally activated *β* relaxation, and with (propensity for) stress-activated shear transformations. Eighth and finally, on the one hand the *υ*_flex,*i*_ of atoms is directly determined by local topological and chemical environment, making the local average a prognostic parameter in monitoring the inherent structural inhomogeneity distributed inside an MG, and on the other hand *υ*_flex_ exhibits robust correlations with local dynamic properties, signalling a structural mechanism to connect with the spatial elastic or plastic heterogeneity^25^,^44^,^53^,^54^,^55^,^56^. As such, the flexibility volume also serves as a quantitative benchmark for explaining the mechanical heterogeneities in MGs. All these attributes make *υ*_flex_ a useful property-revealing indicator of the structural state. In comparison, the frequently invoked free volume (or Ω_a_) is deficient in each of these respects, as illustrated with examples throughout the main text and SI of this paper. In the meantime, the simple *υ*_flex_ is particularly convenient for integration into mathematical equations for theory and modelling, to represent the structural state from local atomic configurations all the way to the global MG sample (system average). All these justify our introduction of the flexibility volume for dealing with MG problems, and incentivize the adoption of this new structural parameter, in lieu of the widely cited but ambiguous free volume, to explain the effective atomic flexibility beyond the traditional space-centric view.

The flexibility volume parameter builds a bridge between the structure and properties of MGs, making the correlation universally quantitative, which was not possible with any of the previous structural indicators. The correlation demonstrated for MGs is derived based on a solid-state physics principle, with no fitting parameters. Our data confirmed that the relationship is not only quantitative, but also indicated that it is universally applicable to various amorphous states of MGs regardless of their composition and processing history. The ability to predict and compare the properties of various MGs based on a single parameter will be interesting to experimentalists who take an MG to different configurational states via thermomechanical processing, in particular intentional rejuvenation of the MG structure^57^, as well as to modellers that need such a quantitative indicator to represent the state the MG is in (as well as the distribution of inhomogeneity inside the glass structure) when writing mathematical equations^8^,^16^,^17^,^58^. Our findings thus address a pressing challenge facing materials scientists in the field of amorphous metals, that is, the lack of robust, causal and mathematically derivable relationships that link the MG structure with properties.

## Methods

### MG samples preparation by MD simulation

Molecular dynamics simulations^59^ have been employed to prepare and analyse the MG models in Supplementary Table 1, using optimized embedded atom method potentials, as performed in our recent publications^40^,^41^,^42^,^43^ and Kob-Andersen LJ (Lennard-Jones) potentials^60^. The samples were quenched to room temperature (300 K) from equilibrium liquids above the corresponding melting points. The quenching was performed using a Nose-Hoover thermostat with zero external pressure. Periodic boundary conditions were applied in all three directions during MD simulation^59^. Voronoi tessellation analysis was employed to investigate the short-range order and atomic volume (Ω_a,*i*_) based on nearest neighbour atoms determined for the MG inherent structure^9^.

### Calculation of vibrational MSD and vibrational anisotropy

In MD simulation, each sample was kept at equilibrium under a microcanonical ensemble (NVE) at room temperature to calculate the vibrational MSD. The MSD of the *i*th atom is defined as: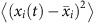, while 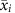 is the equilibrium position of the *i*th atom and the corresponding vibrational MSD obtained on short time scales when the MSD is flat with time and contains the vibrational but not the diffusional contribution. The calculated MSD was taken over 100 independent runs, all starting from the same configuration but with momenta assigned randomly from the appropriate Maxwell-Boltzmann distribution^32^,^33^. The vibrational anisotropy (*η*_*i*_) of the *i*th atom is calculated by monitoring the time-dependent 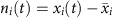, where *n*_*i*_(*t*) is the Euclidean vector to describe the corresponding atomic vibration. Then *η*_*i*_ is measured akin to the definition of structural anisotropy in ref. 61, by averaging the fabric tensor 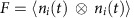, which has three eigenvalues, *λ*_*i*_(1<*i*<3), then 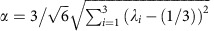. For the isotropic case, *α*=0, while full anisotropy corresponds to *α*=1.

### Energy barrier of thermally activated events

To explore the local PEL (the potential energy minima and the saddle points), we employed the ART nouveau^46^,^47^,^48^. To study the local excitations of the system, initial perturbations in ART were introduced by applying random displacement on a small group of atoms (an atom and its nearest-neighbours). The magnitude of the displacement was fixed, while the direction was randomly chosen. When the curvature of the PEL was found to overcome the chosen threshold, the system was pushed towards the saddle point using the Lanczos algorithm. The saddle point is considered to be found when the overall force of the total system is below 0.01 eVÅ^–1^. The corresponding activation energy is thus the difference between the saddle point energy and the initial state energy. For each group of atoms, we employed ∼100 ART searches with different random perturbation directions. Since there were at least 10,000 such groups in each of our models, more than one million searches by ART were generated in total. After removing the failed searches and redundant saddle points, ∼200,000 different activations, on an average, were identified for each of the samples.

### Data availability

The data that support the findings of this study are available from the corresponding author on request.

## Additional information

**How to cite this article:** Ding, J. *et al*. Universal structural parameter to quantitatively predict metallic glass properties. *Nat. Commun.* **7,** 13733 doi: 10.1038/ncomms13733 (2016).

**Publisher's note**: Springer Nature remains neutral with regard to jurisdictional claims in published maps and institutional affiliations.

## Supplementary Material

Supplementary Information Supplementary Figures 1-11, Supplementary Table 1, Supplementary Notes 1-2 and Supplementary References

Supplementary Movie 1 An example of the vibration of an atomic motif. This atomic motif is from the Cu_64_Zr_36_ MG (Sample G1) at 300 K. The vibrational trajectories of the center atom (marked grey) is tracked and used to determine the local value of flexibility volume for this atom.

Peer Review

## References

[b1] Greer A. L. in *Physical Metallurgy* 5th edn (eds Laughlin, D. E. & Hono, K.) 305–385 (Elsevier, 2014).

[b2] Schroers J. Bulk metallic glasses. Phys. Today 66, 32–37 (2013).

[b3] Yu H. B., Wang W. H. & Samwer K. The β relaxation in metallic glasses: an overview. Mater. Today 16, 183–191 (2013).

[b4] Schuh C. A., Hufnagel T. C. & Ramamurty U. Mechanical behavior of amorphous alloys. Acta Mater. 55, 4067–4109 (2007).

[b5] Chen M. W. Mechanical behavior of metallic glasses: microscopic understanding of strength and ductility. Annu. Rev. Mater. Res. 38, 445–469 (2008).

[b6] Demetriou M. D. . A damage tolerant glass. Nat. Mater. 10, 123–128 (2011). 21217693 10.1038/nmat2930

[b7] Johnson W. L., Demetriou M. D., Harmon J. S., Lind M. L. & Samwer K. Rheology and ultrasonic properties of metallic glass-forming liquids: a potential energy landscape perspective. MRS Bull. 32, 644–650 (2007).

[b8] Falk M. L. & Langer J. S. Dynamics of viscoelastic deformation in amorphous solids. Phys. Rev. E 57, 6 (1998).

[b9] Cheng Y. Q. & Ma E. Atomic-level structure and structure-property relationship in metallic glasses. Prog. Mater. Sci. 56, 379–473 (2011).

[b10] Taylor G. I. & Quinney H. The latent energy remaining in a metal after cold working. Proc. R. Soc. London Series A 143, 307–326 (1934).

[b11] Hirata A. . Direct observation of local atomic order in a metallic glass. Nat. Mater. 10, 28–33 (201). 21102454 10.1038/nmat2897

[b12] Park K. W., Jang J. I., Wakeda M., Shibutani Y. & Lee J. C. Atomic packing density and its influence on the properties of Cu–Zr amorphous alloys. Scr. Mater. 57, 805–808 (2007).

[b13] Wu Y. . Inherent structure length in metallic glasses: simplicity behind complexity. Sci. Rep. 5, 12137 (2015). 26245801 10.1038/srep12137 PMC4642538

[b14] Na J. H. . Compositional landscape for glass formation in metal alloys. Proc. Natl Acad. Sci. USA 111, 9031 (2014). 24927600 10.1073/pnas.1407780111 PMC4078826

[b15] Cohen M. H. & Grest G. Liquid-glass transition, a free-volume approach. Phys. Rev. B 20, 1077 (1979).

[b16] Spaepen F. Microscopic mechanism for steady-state inhomogeneous flow in metallic glasses. Acta Metall. 25, 407–415 (1977).

[b17] Haxton T. K. & Liu A. J. Activated dynamics and effective temperature in a steady state sheared glass. Phys. Rev. Lett. 99, 19 (2007). 10.1103/PhysRevLett.99.195701 18233084

[b18] Kumar G., Neibecker P., Liu Y. H. & Schroers J. Critical fictive temperature for plasticity in metallic glasses. Nat. Commun. 4, 1536 (2011). 10.1038/ncomms2546 PMC3586724 23443564

[b19] Ma E. Tuning order in disorder. Nat. Mater. 14, 547–552 (2015). 25990900 10.1038/nmat4300

[b20] Egami T. Atomic level stresses. Prog. Mater. Sci. 56, 637–653 (2011).

[b21] Kirkpatrick T. R., Thirumalai D. & Wolynes P. G. Scaling concepts for the dynamics of viscous liquids near an ideal glassy state. Phys. Rev. A 40, 1045–1054 (1989). 10.1103/physreva.40.1045 9902230

[b22] Yavari A. R. . Excess free volume in metallic glasses measured by X-ray diffraction. Acta Mater. 53, 1611–1619 (2005).

[b23] Widmer-Cooper A. & Harrowell P. Free volume cannot explain the spatial heterogeneity of Debye–Waller factors in a glass-forming binary alloy. J. Non-Cryst. Solids 352, 5098–5102 (2006).

[b24] Manning M. L. & Liu A. J. Vibrational modes identify soft spots in a sheared disordered packing. Phys. Rev. Lett. 107, 108302 (2011). 21981537 10.1103/PhysRevLett.107.108302

[b25] Ding J., Patinet S., Falk M. L., Cheng Y.Q. & Ma E. Soft spots and their structural signature in a metallic glass. Proc. Natl Acad. Sci. USA 111, 14052 (2014). 25228762 10.1073/pnas.1412095111 PMC4191783

[b26] Johnson W. L & Samwer K. A universal criterion for plastic yielding of metallic glasses with a (T/Tg)2/3 temperature dependence. Phys. Rev. Lett. 95, 195501 (2005). 16383993 10.1103/PhysRevLett.95.195501

[b27] Wang W. H. The elastic properties, elastic models and elastic perspectives of metallic glasses. Prog. Mater. Sci. 57, 487–656 (2012).

[b28] Wang W. H., Wen P., Zhao D. Q., Pan M. X. & Wang R. J. Relation between glass transition temperature and Debye temperature in bulk metallic glasses. J. Mater. Res. 18, 2747–2751 (2006).

[b29] Garrett G. R., Demetriou M. D., Launey M. E. & Johnson W. L. Thermodynamic origin of embrittlement in metallic glasses. Proc. Natl Acad. Sci. USA 113, 10257 (2016). 27573817 10.1073/pnas.1610920113 PMC5027437

[b30] Novikov V.N. & Sokolov A. P. Poisson's ratio and the fragility of glass-forming liquids. Nature 431, 961 (2004). 15496916 10.1038/nature02947

[b31] Krausser J., Samwer K. & Zaccone A. Interatomic repulsion softness directly controls the fragility of supercooled metallic melts. Proc. Natl Acad. Sci. USA 112, 13762 (2015). 26504208 10.1073/pnas.1503741112 PMC4653154

[b32] Widmer-Cooper A., Harrowell P. & Fynewever H. How reproducible are dynamic heterogeneities in a supercooled liquid? Phys. Rev. Lett. 93, 135701 (2004). 15524735 10.1103/PhysRevLett.93.135701

[b33] Widmer-Cooper A. & Harrowell P. Predicting the long-time dynamic heterogeneity in a supercooled liquid on the basis of short-time heterogeneities. Phys. Rev. Lett. 96, 185701 (2006). 16712373 10.1103/PhysRevLett.96.185701

[b34] Mosayebi M., Ilg P., Widmer-Cooper A. & Del Gado E. Soft modes and nonaffine rearrangements in the inherent structures of supercooled liquids. Phys. Rev. Lett. 112, 105503 (2014). 24679306 10.1103/PhysRevLett.112.105503

[b35] Buchenau U., Zorn R. & Ramos M. A. Probing cooperative liquid dynamics with the mean square displacement. Phys. Rev. E 90, 042312 (2014). 10.1103/PhysRevE.90.042312 25375499

[b36] Larini L., Ottochian A., De Michele C. & Leporini D. Universal scaling between structural relaxation and vibrational dynamics in glass-forming liquids and polymers. Nat. Phys. 4, 42 (2007).

[b37] Dyre J. C., Christensen T. & Olsen N. B. Elastic models for the non-Arrhenius viscosity of glass-forming liquids. J. Non-Cryst. Solids 353, 4635–4642 (2006).

[b38] Yu H. B., Richert R., Maaß R. & Samwer K. Unified criterion for temperature-induced and strain-driven glass transitions in metallic glass. Phys. Rev. Lett. 115, 135701 (2015). 26451567 10.1103/PhysRevLett.115.135701

[b39] Chakravarty C., Debenedetti P. G. & Stillinger F. H. Lindermann measure for the solid-liquid phas transition. J. Chem. Phys. 126, 204508 (2007). 17552779 10.1063/1.2737054

[b40] Cheng Y. Q., Ma E. & Sheng H. W. Atomic level structure in multicomponent bulk metallic glass. Phys. Rev. Lett. 102, 245501 (2009). 19659024 10.1103/PhysRevLett.102.245501

[b41] Ding J., Cheng Y. Q. & Ma E. Charge-transfer-enhanced prism-type local order in amorphous Mg_65_Cu_25_Y_10_: short-to-medium-range structural evolution underlying liquid fragility and heat capacity. Acta Mater. 61, 3130–3140 (2013).

[b42] Ding J., Cheng Y. Q., Sheng H. W. & Ma E. Short-range structural signature of excess specific heat and fragility of metallic-glass-forming supercooled liquids. Phys. Rev. B 85, 060201 (2012).

[b43] Zhong L., Wang J., Sheng H. W., Zhang Z. & Mao S. X. Formation of monatomic metallic glasses through ultrafast liquid quenching. Nature 512, 177–180 (2014). 25119235 10.1038/nature13617

[b44] Cheng Y. Q. & Ma E. Configurational dependence of elastic modulus of metallic glass. Phys. Rev. B 80, 064104 (2009).

[b45] Ding J., Cheng Y. Q. & Ma E. Full icosahedra dominate local order in Cu64Zr36 metallic glass and supercooled liquid. Acta Mater. 69, 343–354 (2014).

[b46] Malek R. & Mousseau N. Dynamics of Lennard-Jones clusters: a characterization of the activation-relaxation technique. Phys. Rev. E 62, 7723 (2000). 10.1103/physreve.62.7723 11138044

[b47] Rodney D. & Schuh C. Distribution of thermally activated plastic events in a flowing glass. Phys. Rev. Lett. 102, 235503 (2009). 19658948 10.1103/PhysRevLett.102.235503

[b48] Fan Y., Iwashita T. & Egami T. How thermally activated deformation starts in metallic glass. Nat. Commun. 5, 5083 (2014). 25248915 10.1038/ncomms6083

[b49] Maloney C. E. & Lemaître A. Amorphous systems in athermal, quasistatic shear. Phys. Rev. E 74, 016118 (2006). 10.1103/PhysRevE.74.016118 16907162

[b50] Mattern N. . Structural behavior of Pd_40_Cu_30_Ni_10_P_20_ bulk metallic glass below and above the glass transition. Appl. Phys. Lett. 82, 2589–2591 (2003).

[b51] Mattern N., Bednarcik J., Stoica M. & Eckert J. Temperature dependence of the short-range order of CuZr metallic glass. Intermetallics 32, 51 (2013).

[b52] Suck J. B. Dependence of the atomic dynamics of metallic glasses on quenched-in density fluctuations and on temperature. J. Non-Cryst. Solids 370, 293–295 (2001).

[b53] Greer A. L., Cheng Y. Q. & Ma E. Shear bands in metallic glasses. Mater. Sci. Eng. R 74, 71–132 (2013).

[b54] Wagner H. . Local elastic properties of a metallic glass. Nat. Mater. 10, 439–442 (2011). 21602807 10.1038/nmat3024

[b55] Ding J., Cheng Y. Q. & Ma E. Correlating local structure with inhomogeneous elastic deformation in a metallic glass. Appl. Phys. Lett. 101, 121917 (2012).

[b56] Ma E. & Ding J. Tailoring structural inhomogeneities in metallic glasses to enable tensile ductility at room temperature. Mater. Today http://dx.doi.org/10.1016/j.mattod.2016.04.001 (2016).

[b57] Ketov S. V. . Rejuvenation of metallic glasses by non-affine thermal strain. Nature 524, 200–203 (2015). 26268190 10.1038/nature14674

[b58] Argon A. S. Plastic deformation in metallic glasses. Acta Metall. 27, 47–58 (1979).

[b59] Allen M. P. & Tildesley D. J. Computer Simulation of Liquids Clarendon Press (1987).

[b60] Kob W. & Andersen H. C. Testing mode-coupling theory for a supercooled binary Lennard-Jones mixture I: the van Hove correlation function. Phys. Rev E 51, 4626 (1995). 10.1103/physreve.51.4626 9963176

[b61] Rountree C. L., Vandembroucq D., Talamali M., Bouchaud E. & Roux S. Plasticity-induced structural anisotropy of silica glass. Phys. Rev. Lett. 102, 195501 (2009). 19518968 10.1103/PhysRevLett.102.195501

