# Supplementary material for: Universal structural parameter to quantitatively predict metallic glass properties
Source: Nat Commun. 2016 Dec 12;7:13733. doi: 10.1038/ncomms13733 (PMC5159863; doi:10.1038/ncomms13733)
Supplement: Supplementary Information — Supplementary Figures 1-11, Supplementary Table 1, Supplementary Notes 1-2 and Supplementary References [file ncomms13733-s1.pdf]

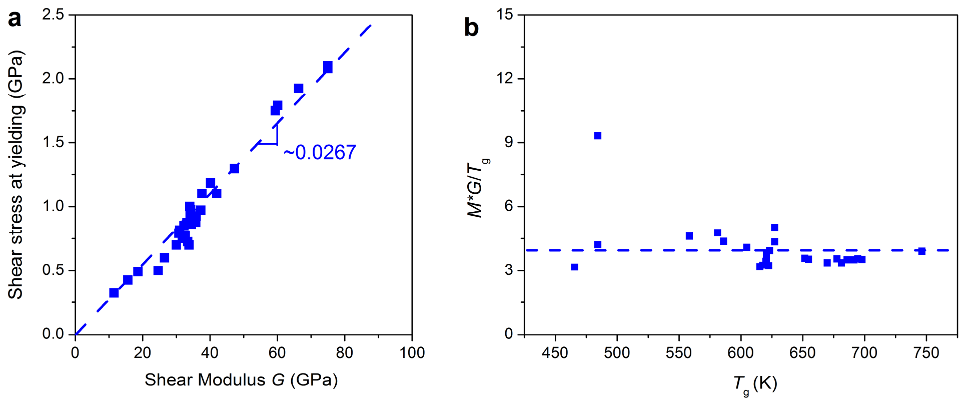

Supplementary Figure 1. Empirical correlation with shear modulus *G* in MGs (a) Experimental yield stress (shear stress at yielding) versus shear modulus *G* at room temperature, for bulk metallic glasses (data extracted from ref. 1 in the supplementary reference list below), manifesting a linear relationship between strength and *G*. (b) The correlation of the glass transition temperature (T_g) with *G* of bulk metallic glasses in experiments (data extracted from ref. 2). *M* is the average atomic mass.

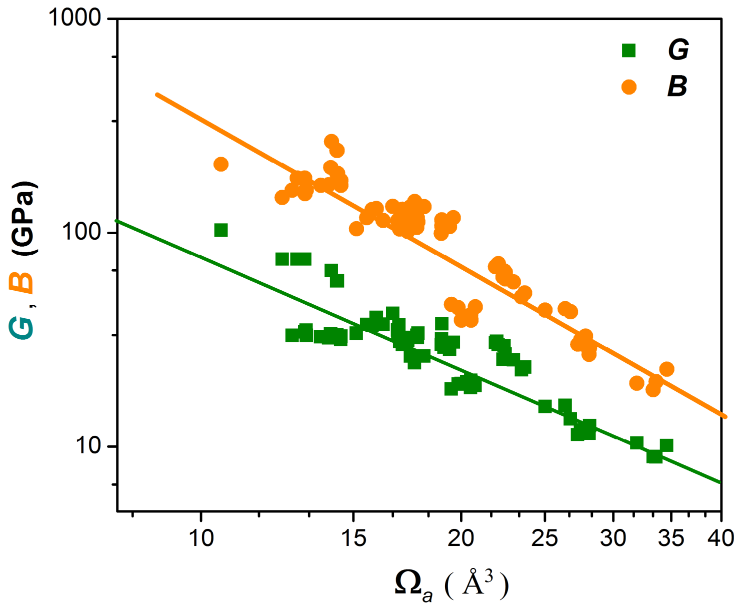

Supplementary Figure 2. Correlation between elastic moduli and atomic volume of MGs. Experimentally measured shear modulus (*G*, as well as bulk modulus, *B*) plotted against sample-averaged atomic volume (Ω_a) in metallic glasses (Data are from the literature[1,3-5]). Note the large data scatter even though the plot is on a logarithmic scale. The straight lines are only meant to show a general trend.

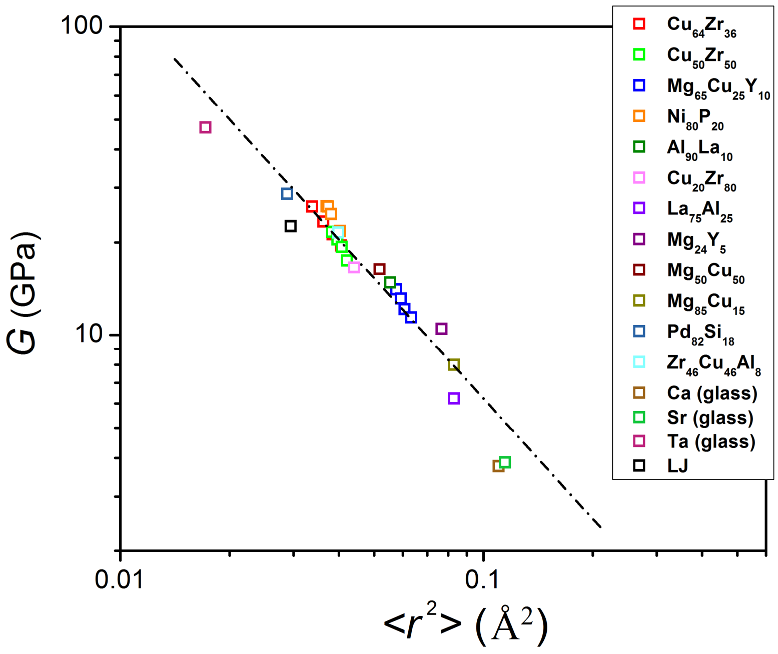

Supplementary Figure 3. Correlation between sample-averaged $\langle r^2 \rangle$ and *G*. $\langle r^2 \rangle$ is the sample-averaged vibrational mean square displacement and *G* is shear modulus, for all the metallic glasses in Supplementary Table 1 (including LJ glass) studied in the present MD simulations. The data scatter is still obvious, even when plotted on a double-log scale.

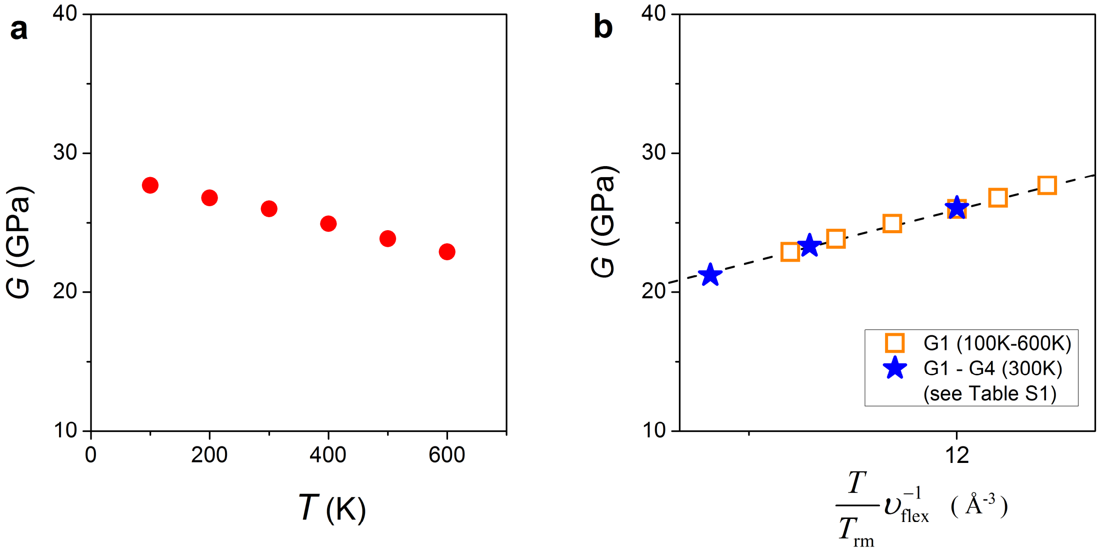

Supplementary Figure 4. Temperature dependence of shear modulus correlated with flexibility volume. (a) Temperature-dependent shear modulus (G) computed for a $Cu_{64}Zr_{36}$ MG prepared at the cooling rate of 10^9 Ks^{-1} (Sample G1 in Supplementary Table 1). The decrease in G with temperature was found to be consistent with the Debye-Grüneisen effect for lowering of the vibrational frequencies (softening of the modes) due to changes in volume (thermal expansion); (b) Relationship between G and temperature-normalized flexibility volume for Sample G1, where T_{rm} is room temperature (300 K) and υ_{flex} is the sample-averaged flexibility volume. The data for G1-G4 ($Cu_{64}Zr_{36}$ MGs with various cooling rates) at 300 K from Fig. 2 in the main text are also included for comparison, to demonstrate that all the data points collapse onto the same linear dependence, when plotted *vs*. a T-normalized flexibility volume. Fitting a straight line (dashed) to the data that is only 7% off the prediction of Eq. (2), $G = C\frac{k_B T}{\upsilon_{flex}} = C \cdot k_B \cdot T_{rm} \cdot (\frac{T}{T_{rm}} \frac{1}{\upsilon_{flex}})$. This plot verifies quantitatively that Eq. (2) is indeed valid for all temperatures below T_g.

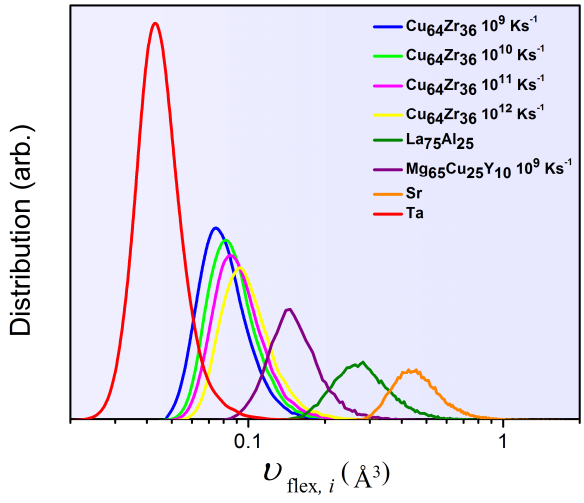

Supplementary Figure 5. Distributions of flexibility volume ($\upsilon_{\mathrm{flex},i}$) for several representative MGs. The data shown in this plot include Cu-Zr, La-Al, Mg-Cu-Y, Sr and Ta MGs studied by MD simulation (see Supplementary Table 1), spanning across two orders of magnitude.

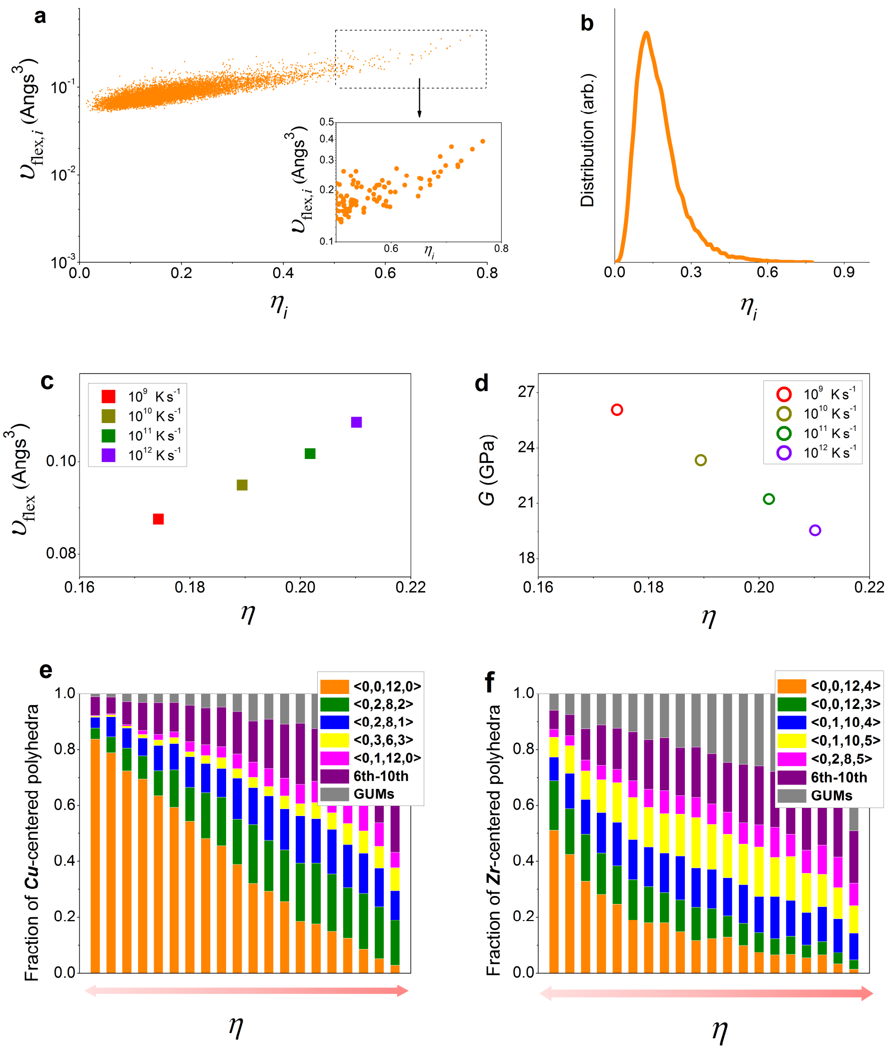

Supplementary Figure 6. Analysis of vibrational anisotropy. (a) Correlation between flexibility volume ($\upsilon_{flex,i}$) and vibrational anisotropy (η_i) for each atom in $Cu_{64}Zr_{36}$ MG (Sample G1). The inset is a magnified view showing that the atoms with the highest value of η_i have the highest value of $\upsilon_{flex,i}$; (b) Corresponding distribution of vibrational anisotropy (η) (Sample G1). For $Cu_{64}Zr_{36}$ MGs with different cooling rates (Sample G1-G4), a strong correlation is observed between the average vibrational anisotropy (η_i) with (c) average flexibility volume (average $\upsilon_{flex,i}$) and (d) shear modulus (G), indicating that large vibrational anisotropy and structural disorder of local atomic environments promote flexibility. To correlate with packing topology, we also show that atoms at the center of different types of (e) Cu-centered and (f) Zr-centered coordination polyhedra exhibit different oscillation anisotropy in $Cu_{64}Zr_{36}$ MG (Sample G1). Each solid bar contains 5% of all the Cu (or Zr) atoms; from left to right, the bins are ordered from the lowest to the highest vibrational anisotropy.

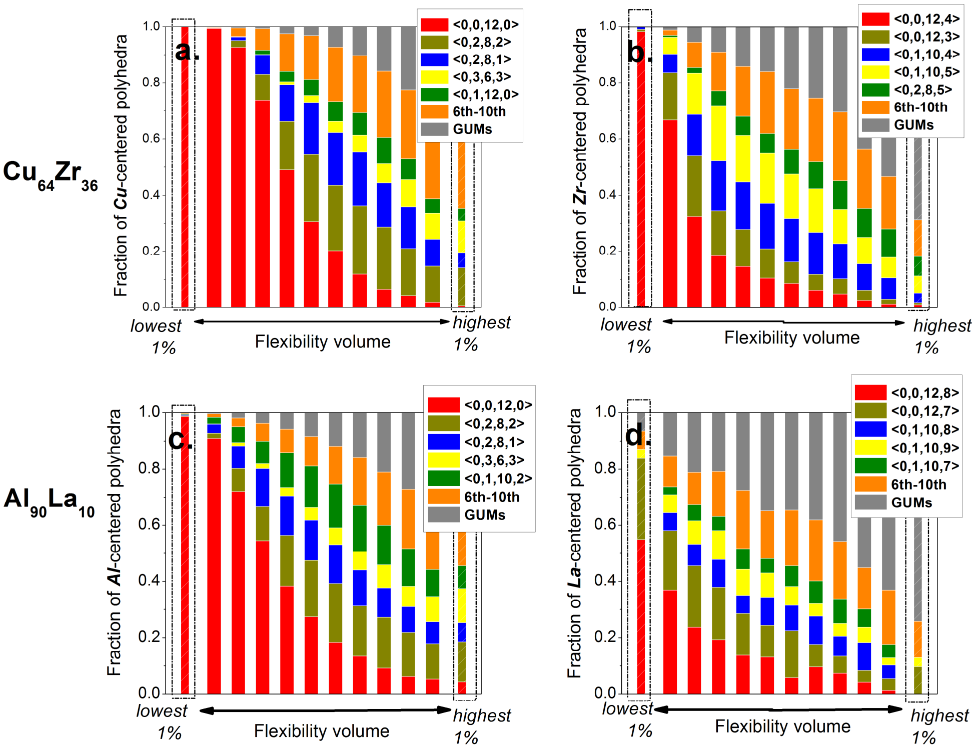

Supplementary Figure 7. Flexibility volume correlates strongly with local atomic packing structure. Atoms at the center of different types of (a) Cu-centered and (b) Zr-centered coordination polyhedra in $Cu_{64}Zr_{36}$ MG (Sample G1), and (c) Al-centered and (d) La-centered coordination polyhedra in $Al_{90}La_{10}$ MG (Sample G17) contribute differently to flexibility volume ($\upsilon_{\mathrm{flex},i}$). Each solid bar contains 10% of all the Cu (or Zr, Al, La) atoms; from left to right, the bins are ordered from the lowest to the highest flexibility volume. Two additional bars contrasting the lowest 1% and the highest 1% of the values of the flexibility volume are drawn to further highlight how topological ordering affects the flexibility volume as defined.

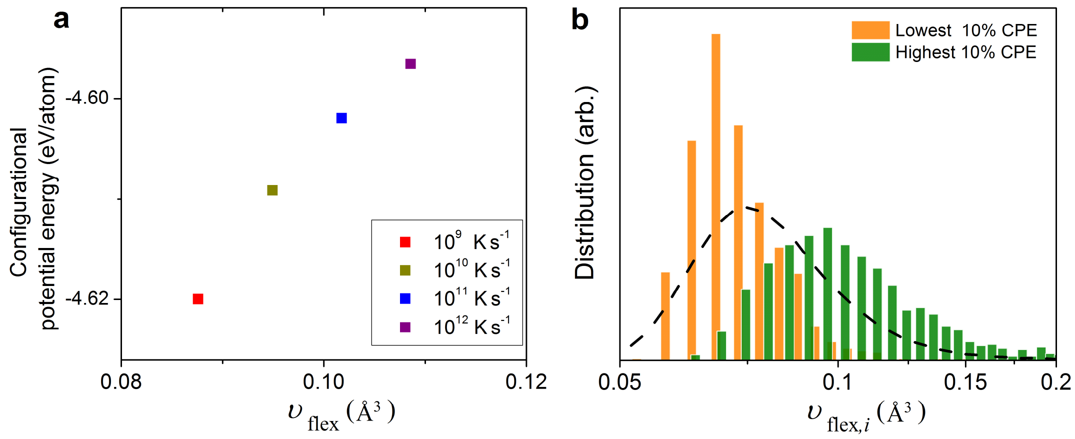

Supplementary Figure 8. Potential energy versus flexibility volume. (a) Ensemble-averaged flexibility volume (υ_{flex}) and configurational potential energy (CPE) for $Cu_{64}Zr_{36}$ metallic glasses obtained with various cooling rates (Sample G1-G4 in Table S1); CPE is the potential energy for the corresponding inherent structure of the metallic glass. (b) Distribution of flexibility volume $\upsilon_{\mathrm{flex},i}$ for atoms with the highest and the lowest configurational potential energy in a $Cu_{64}Zr_{36}$ metallic glass (Sample G1).

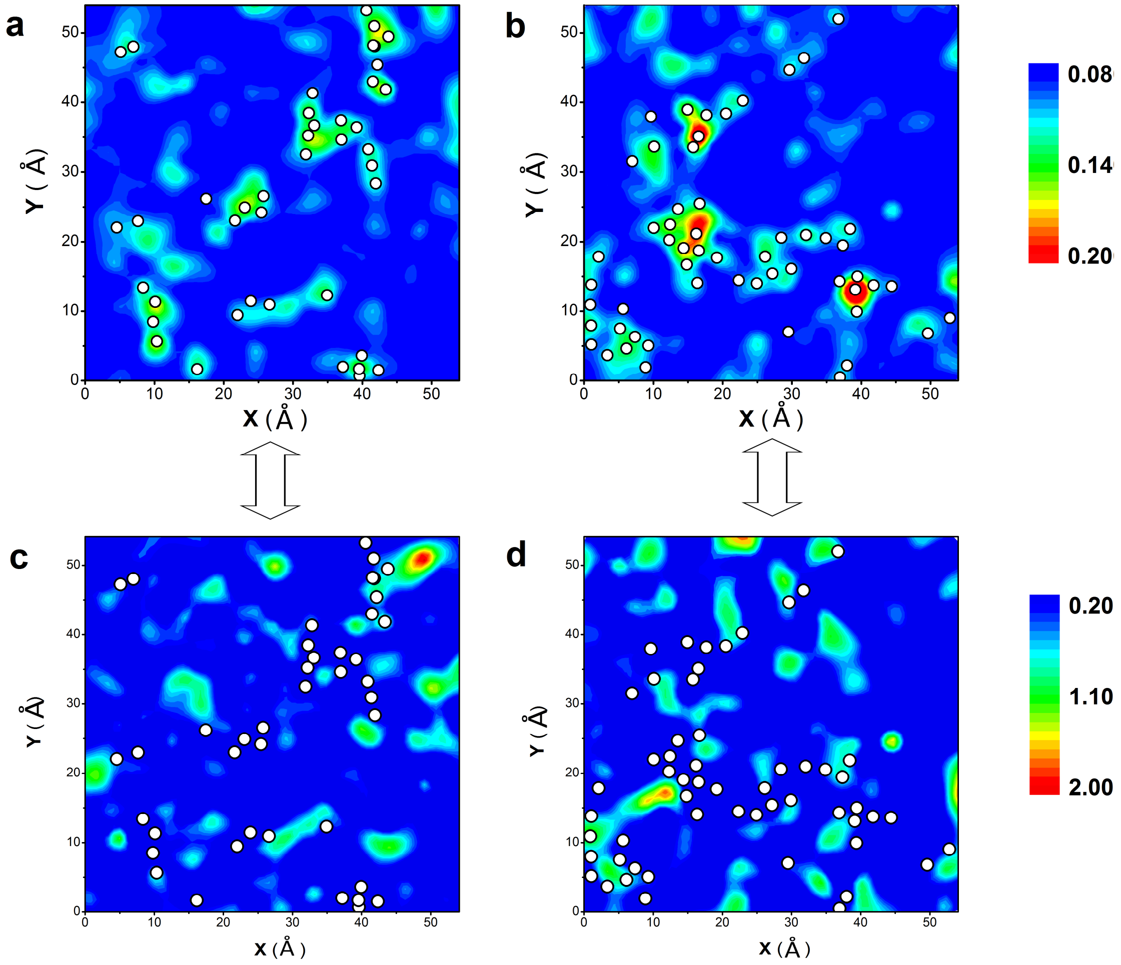

Supplementary Figure 9. Soft spots with strong correlation with local flexibility volume. Soft spots show strong correlation with flexibility volume in (a-b), but little correlation with the variation of local atomic volume in (c-d). The sample is a $Cu_{64}Zr_{36}$ metallic glass (Sample G28). (a-b) Contour maps showing the spatial distribution of flexibility volume $\upsilon_{\text{flex},i}$ (see sidebar). The two sampled representative thin slabs (a-b) each has a thickness of 2.5 Å. White circles superimposed in the maps mark the locations of soft spots (defined as the top 10% of the atoms with the highest participation fraction in low-frequency vibration modes); (c-d) Contour maps showing the spatial distribution of the variation of the local atomic volume (see sidebar for the relative scale); in the plot, to better reflect the variation of the local atomic volume, the latter is depicted in terms of its difference from the average atomic volume of the particular species (e.g. Cu) in the sample. The thin slabs (c-d) are the same as those in (a-b), with white circles representing the locations of soft spots.

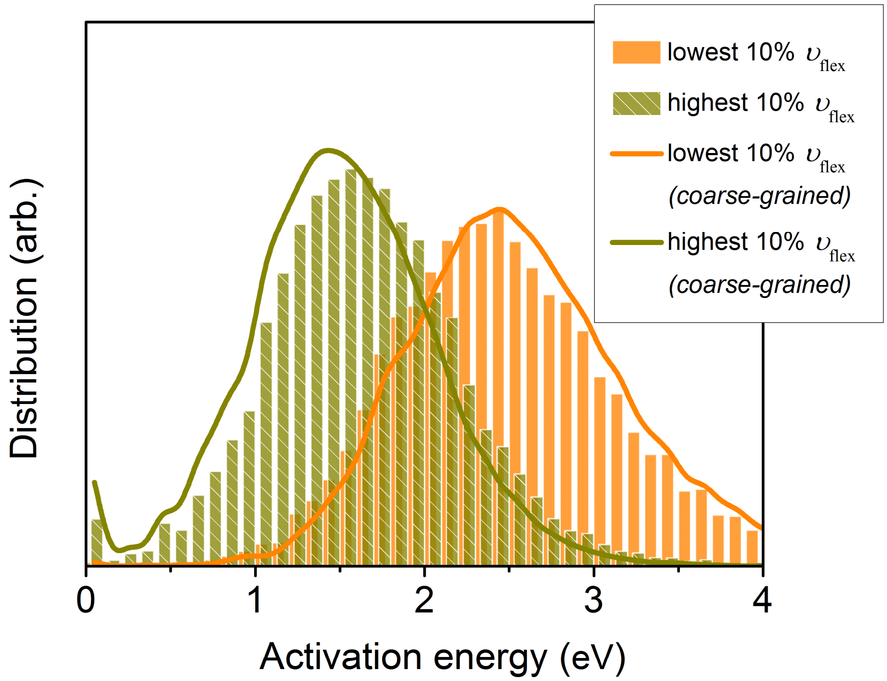

Supplementary Figure 10. **Coarse-grained flexibility volume with strong correlation with activation energy for thermally activated relaxation.** Distribution of activation energy for thermally activated relaxation (correspond to β process) in $Cu_{64}Zr_{36}$ metallic glass (Sample G28) characterized by the activation-relaxation technique (ART nouveau), for central atoms with the highest and the lowest 10% atomic flexibility volume ($\upsilon_{flex,i}$) and the mean flexibility volume of atoms within the first atomic shell. This latter coarse-graining procedure (i.e. averaging over the central atom and its nearest-neighbors) takes into account the fact that thermally activated plastic events usually involve a small group of atoms (*e.g.*, a dozen) rather than one single atom. This procedure is seen to produce an even wider gap between the two peaks of the two groups (peak positions are separated by ~1.0 eV, see solid envelopes), when compared with the cases of atomic $\upsilon_{flex,i}$ (histograms).

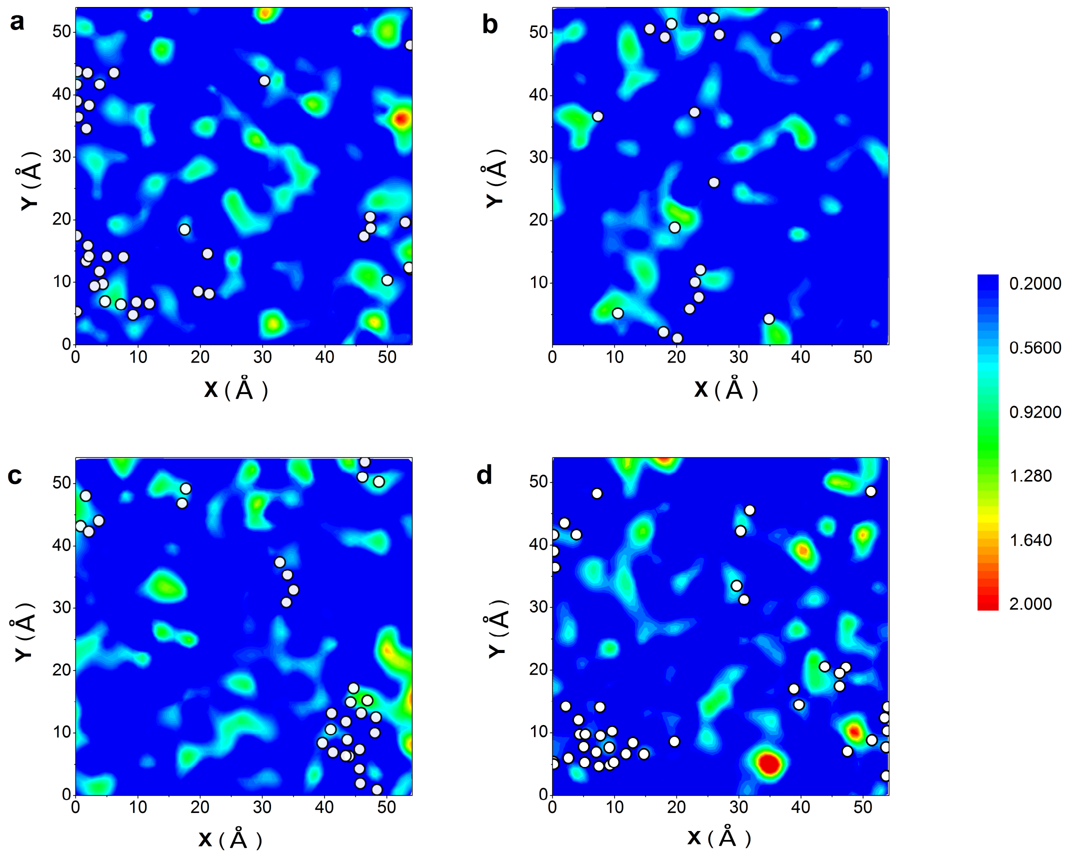

Supplementary Figure 11. Weak correlation between local atomic volume and shear transformation. Contour maps showing the spatial distribution of the variation of local atomic volume (see sidebar for the relative scale) in the $Cu_{64}Zr_{36}$ metallic glass (Sample G28). To better observe the variation of local atomic volume, the latter is depicted in terms of its difference from the average atomic volume of the particular species (Cu or Zr) in the sample. Four slabs (a-d) are sampled for illustration purposes and each has a thickness of 2.5 Å. White circles superimposed in the maps mark the locations of atoms that have experienced the most (top 5%) accumulative non-affine displacement (D^2_{min}), upon athermal quasi-static shear of the simulation box to a global strain of 5%. There is no obvious correlation in this case (in contrast with the strong correlation in the case of $\upsilon_{\mathrm{flex},i}$, Fig. 5 of the main text).

Supplementary Table 1. Metallic glasses studied using molecular dynamics simulations at 300 K: their compositions, number of atoms (*N*), cooling rates and the computed shear modulus (*G*), bulk modulus (*B*) and atomic volume (Ω_a), and the sources of the classical potentials.

#	MGs	*N*	Cooling rate (Ks^{-1})	*G* (GPa)	*B* (GPa)	Ω_a (Å^3/atom)	*Emprical potential*
G1	$Cu_{64}Zr_{36}$	32,000	10^9	26.058	113.83	15.904	ref. 6
G2	$Cu_{64}Zr_{36}$	32,000	10^{10}	23.334	112.72	15.957	ref. 6
G3	$Cu_{64}Zr_{36}$	32,000	10^{11}	21.225	112.16	15.975	ref. 6
G4	$Cu_{64}Zr_{36}$	32,000	10^{12}	19.545	111.62	15.999	ref. 6
G5	$Cu_{50}Zr_{50}$	31,250	10^9	21.599	105.84	17.541	ref. 6
G6	$Cu_{50}Zr_{50}$	31,250	10^{10}	20.416	105.43	17.554	ref. 6
G7	$Cu_{50}Zr_{50}$	31,250	10^{11}	19.344	104.95	17.564	ref. 6
G8	$Cu_{50}Zr_{50}$	31,250	10^{12}	17.500	104.25	17.583	ref. 6
G9	$Mg_{65}Cu_{25}Y_{10}$	30,000	10^9	14.056	41.95	20.909	ref. 7
G10	$Mg_{65}Cu_{25}Y_{10}$	30,000	10^{10}	13.118	41.455	20.929	ref. 7
G11	$Mg_{65}Cu_{25}Y_{10}$	30,000	10^{11}	12.121	40.996	20.929	ref. 7
G12	$Mg_{65}Cu_{25}Y_{10}$	30,000	10^{12}	11.400	40.601	20.965	ref. 7
G13	$Ni_{80}P_{20}$	32,000	10^9	26.136	180.55	11.099	ref. 8
G14	$Ni_{80}P_{20}$	32,000	10^{10}	26.057	180.35	11.116	ref. 8
G15	$Ni_{80}P_{20}$	32,000	10^{11}	24.689	178.77	11.124	ref. 8
G16	$Ni_{80}P_{20}$	32,000	10^{12}	21.724	176.69	11.137	ref. 8
G17	$Al_{90}La_{10}$	31,250	10^{10}	14.807	62.433	18.942	ref. 9
G18	$Cu_{20}Zr_{80}$	31,250	10^{10}	16.568	88.811	20.987	ref. 6
G19	$La_{75}Al_{25}$	31,250	10^{11}	6.2204	33.87	31.742	ref. 9
G20	$Mg_{24}Y_5$	58,000	10^{10}	10.46	32.936	25.252	ref. 10
G21	$Mg_{50}Cu_{50}$	31,250	10^{11}	16.367	55.176	16.262	ref. 7
G22	$Mg_{85}Cu_{15}$	31,250	10^{11}	7.996	33.757	21.408	ref. 7
G23	$Pd_{82}Si_{18}$	31,250	10^{10}	28.68	147.29	14.678	ref. 11
G24	$Zr_{46}Cu_{46}Al_8$	31,250	10^{11}	21.491	108.44	17.64	ref. 6
G25	*Amorph.* Ca	31,250	$5\times10^{12*}$	3.627	15.523	43.268	ref. 12
G26	*Amorph.* Sr	31,250	$5\times10^{12*}$	3.9644	11.745	56.305	ref. 12
G27	*Amorph.* Ta	31,250	$5\times10^{12*}$	47.21	168.82	18.936	ref. 13
G28	$Cu_{64}Zr_{36}$	10,000	10^9	26.06	113.8	15.90	ref. 6
G29	$Cu_{64}Zr_{36}$	10,000	10^{10}	23.41	112.8	15.96	ref. 6
G30	$Cu_{64}Zr_{36}$	10,000	10^{11}	21.22	112.2	15.98	ref. 6
G31	$Cu_{64}Zr_{36}$	10,000	10^{12}	19.55	111.6	16.00	ref. 6
G32	LJ ($A_{80}B_{20}$)	16,000	$2\times10^{10\,ǂ}$	22.55^ǂ	82.20^ǂ	10.97^ǂ	ref. 14

*These MGs were quenched to 300K from equilibrium liquids with the denoted cooling rate, and then relaxed at 300K for 50 *ns* with *NPT* ensemble.

ǂ The units for the Kob-Andersen Lennard-Jones (LJ) binary glass are normalized from reduced units.

Supplementary Note 1: Derivation of Eq. (2), the universal υ_{flex} - G relation

Eq. (2) in the main text,

$$G = C\frac{k_{\mathrm{B}}T}{\langle r^2\rangle \cdot a} = C\frac{k_{\mathrm{B}}T}{\upsilon_{\mathrm{flex}}}$$

illustrates that the υ_{flex} as defined deterministically controls shear modulus *G*. This relation stems from the Debye model and can be derived as follows.

The Debye temperature θ_{D} can be expressed as[3,15]:

$$\theta_D = \frac{h}{k_{\mathrm{B}}}\left(\frac{4\pi}{9}\right)^{-1/3}(\Omega_{\mathrm{a}})^{-1/3}\left(\frac{1}{\nu_{\mathrm{l}}^3}+\frac{2}{\nu_{\mathrm{s}}^3}\right)^{-1/3}, \qquad \text{(i)}$$

where ν_{l} ($\nu_{\mathrm{l}} = \sqrt{\frac{B+\frac{4}{3}G}{\rho}}$) and ν_{s} ($\nu_{\mathrm{s}} = \sqrt{\frac{G}{\rho}}$) are the longitudinal and transverse sound velocities, respectively, and $\rho = \frac{m}{\Omega_{\mathrm{a}}}$ is the mass density, where *m* is average atomic weight. For MGs, as known for >100 alloys measured by experiment[3], $\nu_{\mathrm{l}} = 1.8 - 2.5\nu_{\mathrm{s}}$. Approximating $\nu_{\mathrm{l}} = 2\nu_{\mathrm{s}}$ would then only cause an error of no more than ~2% in θ_{D}, since the second term overrides the first term in the last bracket of Eq. (i). Hence, Eq. (i) is simplified to:

$$\theta_{\mathrm{D}} \approx \frac{h}{k_{\mathrm{B}}}\left(\frac{4\pi}{9}\right)^{-1/3}\left(\frac{17}{8}\right)^{-1/3}(\Omega_{\mathrm{a}})^{-1/3}\nu_{\mathrm{s}} = \frac{h}{k_{\mathrm{B}}}\left(\frac{4\pi}{9}\right)^{-1/3}\left(\frac{17}{8}\right)^{-1/3}(\Omega_{\mathrm{a}})^{-1/3}\sqrt{\frac{G}{\rho}}. \qquad \text{(ii)}$$

Now, the Debye temperature is also known to scale with the vibrational MSD, $\left\langle r^2 \right\rangle$, following[16,17] at the high temperature limit:

$$\theta_{\mathrm{D}}{}^2 = \frac{9\hbar^2 T}{m k_{\mathrm{B}} \left\langle r^2 \right\rangle}. \tag{iii}$$

Combining Eqs. (i-iii), we arrive at Eq. (2) given in the main text

$$G == C\frac{k_{\mathrm{B}}T}{\left\langle r^2 \right\rangle \cdot a} = C\frac{k_{\mathrm{B}}T}{\frac{\left\langle r^2 \right\rangle}{a^2} \cdot a^3} = C\frac{k_{\mathrm{B}}T}{\upsilon_{\mathrm{flex}}} \quad ,$$

where $C = \frac{9}{4\pi^2}\left(\frac{4\pi}{9}\right)^{2/3}\left(\frac{17}{8}\right)^{2/3}$ is a universal constant.

This derivation predicts that at a given temperature T (*e.g.*, room temperature), a single indicator, υ_{flex} alone, can predict G for all MGs (previously, the relation between G and MSD was discussed for liquids[18,19]). Clearly, the new flexibility volume indicator υ_{flex} is neither an equivalent substitute of other volume parameters, (Ω_{a}, υ_{f}, etc.), nor a fudge factor in equations. Rather, υ_{flex} is unambiguously defined and a truly property-controlling structural parameter in Eq. (2) of the main text.

Figure 2 in the main text shows the universal relation of υ_{flex} - G for ~32 model MGs at room temperature (300 K). For MGs at various temperatures (below T_{g}) other than room temperature, Eq. (2) also works, as seen in Supplementary Figure 4. In that plot, the X axis is in the form of a temperature normalized flexibility volume, $\frac{T}{T_{\mathrm{rm}}}\upsilon_{\mathrm{flex}}^{-1}$, where T_{rm} is room

temperature, υ_{flex} the ensemble-averaged flexibility volume. At room temperature, $\frac{T}{T_{\text{rm}}}\upsilon_{\text{flex}}^{-1} = \upsilon_{\text{flex}}^{-1}$, as shown in Fig. 2.

Supplementary Note 2: Experimental measurement of the flexibility volume

This section provides information as to how the flexibility volume can be measured experimentally, in addition to the computational analyses reported in the main text. As seen from the equations in the preceding sub-section (derivation), the flexibility volume υ_{flex} can be calculated from the MSD, $\langle r^2 \rangle$, or Debye temperature θ_{D}, both of which can be measured in the laboratory via Inelastic Neutron Scattering (INS)[20-24], Extended X-Ray Absorption Fine Structure (EXAFS)[25,26], and X-ray/neutron diffraction[27-33]. The acoustic method for measuring sound velocities has also been employed to characterize the Debye temperature[3]. In the following we will briefly discuss these methods to illustrate how they can be used to measure the flexibility volume in MGs.

INS is a powerful tool to characterize atomic level dynamics of materials, because thermal neutrons have the desired combination of wavelength (comparable to typical interatomic distances), momentum (comparable to the size of the Brillouin zone), and energy (comparable to the excitation of phonons). INS has been widely used to measure phonon dispersion curves of single crystals, vibrational density of states (VDOS) across a wide range of frequency ω in crystalline and amorphous materials, and even more complicated (such as higher order and magnetic) excitations and dynamical properties. According to the theory of phonons, both the MSD and Debye temperature (and therefore

flexibility volume) can be calculated if the VDOS is known. Specifically, INS measures the dynamical structure factor $S(Q,\omega)$, which is related to the VDOS $g(\omega)$ by[34]:

$$S(Q,\omega)=\frac{Q^2}{4m}\exp(-2W)\frac{g(\omega)}{\omega}[n(\hbar\omega)+1] \quad , \tag{iv}$$

where $\exp(-2W)$ is the Debye-Waller factor, with W proportional to Q^2 and the average MSD (at a given Q and temperature this term is a constant), $n(\hbar\omega)$ is the Bose-Einstein occupation factor, and $g(\omega)$ satisfies $\int g(\omega)d\omega=1$. In an INS experiment one measures $S(Q,\omega)$ at a fixed temperature and a range of Q, such that the Q-integrated intensity as a function of ω scales with $\frac{g(\omega)}{\omega}[n(\hbar\omega)+1]$ by a constant. Then $g(\omega)$ can be obtained through normlization[35].

To extract Debye temperature from $g(\omega)$, we follow the Debye model in which the VDOS in a 3D solid is simplified as $g(\omega)=\frac{3\omega^2}{\omega_D^3}$, where ω_D is Debye frequency and $\hbar\omega_D=k_B\theta_D$. If the true $g(\omega)$ is measured, the Debye temperature can be calculated by fitting the Debye model to the real $g(\omega)$, i.e., $\theta_D=\frac{4}{3}\frac{\langle E\rangle}{k_B}$, where $\langle E\rangle=\int\hbar\omega g(\omega)d\omega$. Alternatively, the MSD can also be directly calculated from $g(\omega)$, following:

$$\langle r^2\rangle=\frac{3\hbar}{m}\int_0^\infty\frac{g(\omega)}{\omega}[n(\hbar\omega)+\frac{1}{2}]d\omega \quad . \tag{v}$$

The above equations suggest that the flexibility volume can be derived from the measured VDOS.

It should be noted that since different elements have different neutron scattering cross-sections, the total VDOS measured by INS is in fact the sum of the partial contribution from each element weighted by its cross-section. For this reason, the VDOS measured by INS is often called generalized VDOS (GVDOS). Depending on the composition and the scattering cross-section contrast, the GVDOS may or may not be the same as the true VDOS. To "undo" this weighting effect, information on the partial VDOS would be needed. This can be done by taking advantage of the fact that for a given element, the neutron scattering cross-section is usually different for different isotopes. This allows isotope labeling which is a widely used technique in neutron scattering to resolve the partial contributions from each element[36]. In certain cases, if the constituent elements of a system have similar neutron scattering cross-sections (*e.g.*, in Cu-Zr), the measured GVDOS should be very close to the true VDOS[37].

A fundamentally different approach to experimentally characterize flexibility volume is by measuring the Debye-Waller factor directly, based on the Debye-Waller effect from atomic vibration. When atoms vibrate, the peak intensity in the diffraction pattern in reciprocal space, or the pair distribution function in real space, will be suppressed, due to uncertainty of the atomic positions. This suppression can be calculated to follow an exponential form $\exp(-2W)$, which is the so-called Debye-Waller factor. The Debye-Waller factor is directly correlated with MSD (the W term is proportional to the MSD, as well as Q^2), and also related to the Debye temperature. Therefore, if one can obtain Debye-

Waller factor by, for example, measuring the sample at different temperatures and monitor how the peak intensity decreases with increasing temperature, the MSD and flexibility volume can then be derived. This principle applies for various techniques such as powder diffraction, total scattering and pair distribution function analysis, and EXAFS.

Powder diffraction has been routinely used to measure MSD in crystals. The intensity of the Bragg peaks decreases as the temperature rises, and the magnitude of the temperature effect is also a function of the momentum transfer, Q. It can be derived that the actual Bragg peak intensity at finite temperature is $I = I_0 \exp(-\frac{1}{2}Q^2 \left\langle r^2 \right\rangle)$, where I_0 is the zero point intensity. Therefore, by plotting the peak intensity as a function of Q and temperature, one can extrapolate to obtain I_0, and then the desired $\left\langle r^2 \right\rangle$ at a given temperature[35]. The Debye-Waller factor is also a parameter that is fitted and optimized in Rietveld refinement (the B factor), and it is an output when such refinement is performed on a crystal structure[38].

Total scattering is useful to study both crystalline and amorphous materials, and it is expecially suitable for the study of nanoscrystalline and amorphous samples where long-range order tends to be weak or absent. The intensity and shape of the peaks in structure factor and pair distribution functions carry information on the interatomic distances and atomic vibration. Specifically, by measuring the structure factor $S(Q)$ at different temperatures, the Debye-Waller factor and MSD can be calculated by taking the ratio[31-33],

$$\frac{S_{T_2}(Q)-1}{S_{T_1}(Q)-1} = \exp\left\{-2\left[W_{T_2}(Q) - W_{T_1}(Q)\right]\right\}. \qquad \text{(vi)}$$

Following the Debye theory, the Debye-Waller factor as a function of Q can be expressed as:

$$W_T = \frac{3\hbar^2 Q^2}{2mk_B\theta_D}\left(\frac{T}{\theta_D}\right)^2 \int_0^{\frac{\theta_D}{T}} \left(\frac{1}{2}+\frac{1}{e^z-1}\right) z dz \qquad , \qquad \text{(vii)}$$

which means that one can solve the Debye temperature by measuring the temperature dependent structure factor[31-33]. A similar procedure has been used to study the real space pair distribution function[39].

In addition to X-ray or neutron diffraction, EXAFS is also a popular technique for structural characterization. It can probe element-specific local atomic environment. The measured EXAFS can be theoretically expressed as[25,26]:

$$\chi(k) = \sum_j \frac{N_j S_0^2}{kR_j^2} \left| f_j^{\text{eff}}(k, R_j) \right| \sin(2kR_j + \varphi_j(k)) e^{-2R_j/\lambda} e^{-2\sigma_j^2 k^2} \qquad , \qquad \text{(viii)}$$

where the sum runs over all unique scattering paths j of degeneracy N_j. $f_j^{\text{eff}}(k, R_j)$ is the effective scattering amplitude, R_j is the half-path length, and σ_j^2 is the mean square variation of R_j. It is clear that in the above equation $e^{-2\sigma_j^2 k^2}$ plays the role of the Debye-Waller factor as in the diffraction equations. However, there are also differences. First, σ_j^2 is the variation of a path, and it is related to not only the MSD of involved atoms, but also the relative phase (*i.e.*, correlation) of the atomic displacements. Second, only the relative motion parallel to the path has an impact – the portion perpendicular to the path is irrelevant. Despite the complexity, theory has been developed to connect the temperature dependence

of EXAFS with the Debye theory[26], allowing measurement of Debye temperature or MSD through this method.

Fundamentally, the flexibility volume, MSD, Debye temperature, and Debye-Waller factor are all correlated, and we can derive one from another within the Debye theory. This offers various techniques (as discussed above) that can be potentially employed to study MGs and their structure-property relationship. In fact, there have been studies showing that Debye temperature is sensitive to the processing history of MGs[28], and this is consistent with our MD simulations of the flexibility volume. One expects MGs of different compositions to have quite different flexibility volumes. Also, for a given MG composition, the processing history makes a difference; a melt spun ribbon would exhibit a larger υ_{flex}, compared with a bulk sample that has been aged and relaxed extensively[28,29]. Although these parameters, except for Debye temperature, can all be defined on an atomic/local level, experimental characterization has been mainly on the macroscopic (sample) level. However, with the development of techniques that probe the local structure/dynamics, such as sub-nanometer-sized electron beam and submicron-sized X-ray beam, measurement of flexibility volume at various microscopic levels is expected to be feasible in the near future.

Supplementary References

1. Johnson, W.L & Samwer, K. A universal criterion for plastic yielding of metallic glasses with a (T/Tg)2/3 temperature dependence, *Phys. Rev. Lett*, **95**, 195501 (2005).

2. Wang, W. H., Wen, P., Zhao, D. Q., Pan, M. X., & Wang, R. J. Relation between glass transition temperature and Debye temperature in bulk metallic glasses, *J. Mater. Res.* **18**, 2747-2751 (2006).

3. Wang, W. H. The elastic properties, elastic models and elastic perspectives of metallic glasses, *Prog. Mater. Sci.* **57**, 487-656 (2012).

4. Zhang, Z., Keppens, V., Senkov, O. N., Miracle, D.B. Elastic properties of Ca-based bulk metallic glasses studied by resonant ultrasound spectroscopy. *Mater. Sci. Eng.: A.* **471**, 151-154 (2007).

5. Wang, W. H. Correlations between elastic moduli and properties in bulk metallic glasses. *J. Appl. Phys*. **99**, 093506 (2006).

6. Cheng, Y. Q., Ma, E., & Sheng, H. W. Atomic level structure in multicomponent bulk metallic glass. *Phys. Rev. Lett.* **102**, 245501 (2009).

7. Ding, J., Cheng, Y. Q., & Ma, E. Charge-transfer-enhanced prism-type local order in amorphous $Mg_{65}Cu_{25}Y_{10}$: Short-to-medium-range structural evolution underlying liquid fragility and heat capacity. *Acta Mater.* **61**, 3130-3140 (2013).

8. Sheng, H. W., Ma, E., & Kramer, M. Relating dynamic properties to atomic structure in metallic glasses. *JOM* **64**, 856-881 (2012).

9. Sheng, H. W. EAM potentials of La-Al. https://sites.google.com/site/eampotentials/Home/LaAl (2011)

10. Sheng, H. W. EAM potentials of Mg-Y. https://sites.google.com/site/eampotentials/Home/MgY (2011)

11. Ding, J., Cheng, Y. Q., Sheng, H. W., & Ma, E. Short-range structural signature of excess specific heat and fragility of metallic-glass-forming supercooled liquids. *Phys. Rev. B* **85**, 060201 (2012).

12. Sheng, H. W., Kramer, M., Cadien, A., Fujita, T., Chen, M. Highly optimized embedded-atom-method potentials for fourteen fcc metals. *Phys. Rev. B* **83**, 134118 (2011).

13. Zhong, L., Wang, J., Sheng, H. W., Zhang, Z., Mao, S. X. Formation of monatomic metallic glasses through ultrafast liquid quenching. *Nature* **512**, 177-180 (2014).

14. Kob, W., & Andersen, H. C. Testing mode-coupling theory for a supercooled binary Lennard-Jones mixture I: The van Hove correlation function. *Phys. Rev E.* **51**, 4626 (1995).

15. Kittel, C. Introduction to solid state physics. 6th ed. New York: John Wiley & Sons, (1986).

16. Lindemann, A. Ueber die Berechnung molekularer Eigenfrequenzen. Z Phys 11: 609(1910).

17. Reisland, J. A. The Physics of Phonons, Wiley, London (1973).

18. Buchenau, U., Zorn, R. & Ramos, M. A. Probing cooperative liquid dynamics with the mean square displacement. *Phys. Rev. E.* **90**, 042312 (2014)

19. Dyre, J. C., Christensen, T. & Olsen, N. B. Elastic models for the non-Arrhenius viscosity of glass-forming liquids. *J. Non-Cryst. Solids.* **353**, 4635-4642 (2006)

20. Holden, T. M., Dugdale, J. S., Hallam, G.C., Pavuna, D. Neutron inelastic scattering measurements on the metallic glass $Cu_{0.6}Zr_{0.4}$. *J. Phys. F: Metal Phys.* **11**, 1737 (1981)

21. Syrykh, G. F., Zhernov, A. P., Chernoplekov, N. A. Soft anharmonic vibrational states in metallic glasses. *Sov. Phys. JETP* **73**, 542 (1981)

22. Caciuffo, R., et al. The generialised vibrational density of states of the metallic glass Fe40Ni40B20 determined by neutron inelastic scattering. *J. Phys. Condens. Matter.* **1**, 5621 (1989).

23. Syrykh, G. F., Zemlyanov, M. G., & Ishmaev, S. N. Atomic vibration spectra of the metallic glass Ni64Zr36. *Phys. Solid State*. **40**, 1 (1998).

24. Otomo, T., Arai, M., Suck, J. B., Bennington, S. M. An experimental approach to reveal the origin of collective excitations in $Ni_{33}Zr_{67}$ metallic glass. *J. Non-Cryst. Solids* **312-314**, 599 (2002).

25. Vila, F. D., Rehr, J. J., Rossner, H. H., Krappe, H. J. Theoretical X-ray absorption Debye-Waller factors, *Phys.Rev. B* **76**, 014301 (2007).

26. Poiarkova, A. V. X-ray absorption fine structure Debye-Waller factors. PhD Thesis, University of Washington (1999).

27. Suck, J. B., Rudin, H., Guntherodt, H. J., Beck, H. Influence of structural relaxation on the atomic dynamics of the metallic glass $Mg_{70}Zn_{30}$. *J. Non-Crystal. Solids* **61&62**, 295 (1984).

28. Suck, J. B. Dependence of the atomic dynamics of metallic glasses on quenched-in density fluctuations and on temperature. *J. Non-Cryst. Solids,* **370**, 293-295 (2001).

29. Suck, J. B. Temperature dependence of the generalized vibrational density of states of melt spun ZrCuAl, *Mater. Sci. Eng. A* **479**, 226-228 (1997).

30. Suck, J. B., Schober, H., Guntherodt, H. The generalized vibrational density of states and total dynamic structure factor of the metallic glass ZrCuNiAl measured at room temperature. *J. Non-Cryst. Solids*. **733**, 205-207 (1996).

31. Mattern, N., Hermann, H., Roth, S., Sakowski, J., Macht, M., Jovari, P., Jiang, J. Z. Structural behavior of PdCuNiP bulk metallic glass below and above the glass transition. *Appl. Phys. Lett*. **82**, 2589 (2003).

32. Mattern, N., Eckert, J., Kuhn, U., Hermann, H., Sakowski, J., Herms, G., Neuefeind, J. Structural behavior of ZrTiCuNiAl bulk metallic glass at high temperatures, *Appl. Phys. Lett*. **80**, 4525 (2002).

33. Mattern, N., Bednarcik, J., Stoica, M., Eckert, J. Temperature dependence of the short-range order of CuZr metallic glass. *Intermetallics* **32**, 51 (2013).

34. Squires, G. L. Introduction to the theory of thermal neutron scattering (1997).

35. Kresch, M., et al. Neutron scattering measurements of phonons in nickel at elevated temperatures, *Phys. Rev. B* **75**, 104301 (2007).

36. Syrykh, G. F., Zemlyanov, M. G., Ishmaev, S. N. Experimental study of partial vibrational spectra in amorphous alloys, *Physica B: Condens Matter* **450**, 234-236, (1997).

37. Ma, J., et al. Glass-like phonon scattering from a spontaneous nanostructure in AgSbTe2, *Nature Nanotech*. **8**, 445-451 (2013).

38. Willis, B. T. M., & Pryor, A. W. Thermal vibrations in crystallography, Cambridge University Press (1975).

39. Schoening, F. R. L. The structure of amorphous silicon telluride, *J. Mater. Sci*. **14**, 2397 (1979).
