# Supplementary material for: Universal structural parameter to quantitatively predict metallic glass properties
Source: Nat Commun. 2016 Dec 12;7:13733. doi: 10.1038/ncomms13733 (PMC5159863; doi:10.1038/ncomms13733)
Supplement: Peer Review [file ncomms13733-s3.pdf]

PEER REVIEW FILE

Editorial Note: this manuscript has been previously reviewed at another journal that is not operating a transparent peer review scheme. This document only contains reviewer comments and rebuttal letters for versions considered at Nature Communications.
